# Artificial Intelligence‐Empowered Automated Double Emulsion Droplet Library Generation

**DOI:** 10.1002/smll.202412099

**Published:** 2025-03-25

**Authors:** Seonghun Shin, Owen D. Land, Warren D. Seider, Jinkee Lee, Daeyeon Lee

**Affiliations:** ^1^ Department of Chemical and Biomolecular Engineering School of Engineering and Applied Science University of Pennsylvania Philadelphia PA 19104 USA; ^2^ School of Mechanical Engineering Sungkyunkwan University Suwon 16419 Republic of Korea; ^3^ Institute of Quantum Biophysics Sungkyunkwan University Suwon 16419 Republic of Korea

**Keywords:** convolutional neural network, experiment automation, feedback control, microfluidics, object detection

## Abstract

Double emulsions with core‐shell structures are versatile materials used in applications such as cell culture, drug delivery, and materials synthesis. A droplet library with precisely controlled dimensions and properties would streamline screening and optimization for specific applications. While microfluidic droplet generation offers high precision, it is typically labor‐intensive and sensitive to disturbances, requiring continuous operator intervention. To address these limitations, we present an artificial intelligence (AI)‐empowered automated double emulsion droplet library generator. This system integrates a convolutional neural network (CNN)‐based object detection model, decision‐making, and feedback control algorithms to automate droplet generation and collection. The system monitors droplet generation every 171 ms—faster than a Formula 1 driver's reaction time—ensuring rapid response to disturbances and consistent production of single‐core double emulsions. It autonomously generates libraries of 25 distinct monodisperse droplets with user‐defined properties. This automation reduces labor and waste, enhances precision, and supports rapid and reliable droplet library generation. We anticipate that this platform will accelerate discovery and optimization in biomedical, biological, and materials research.

## Graphical abstract

An artificial intelligence (AI)‐empowered automated droplet library generator (ADLib) is introduced to streamline droplet generation experiments. By integrating a convolutional neural network (CNN)‐based object detection model with feedback control, it autonomously generates 25 distinct monodisperse double emulsions with user‐specified properties. Operating in real‐time, it ensures precise, rapid, and consistent droplet production, reducing labor, waste, and barriers of the droplet generation.

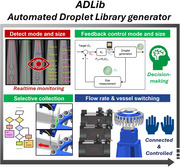

## 1 Introduction

Double emulsions consist of liquid droplets dispersed in other liquid droplets, creating core‐shell structured compound emulsions. Due to their capacity to encapsulate and protect materials, double emulsions have been widely used in scientific and industrial applications.^[^ ^1^ ^]^ For example, they provide an ideal controlled environment for cell culture^[^ ^2^, ^3^, ^4^ ^]^ and bioassays,^[^ ^5^, ^6^ ^]^ and also serve as drug delivery vehicles enabling targeted delivery and controlled release.^[^ ^7^, ^8^, ^9^, ^10^ ^]^ To fully leverage their potential in these applications, the dimensions and properties of double emulsion droplets must be optimized, as slight variations in their structures can lead to significant changes in their efficacy and functionality.^[^ ^11^, ^12^ ^]^ For instance, shell thickness, composition, and droplet size influence the rate and timing of drug release from the drug delivery systems,^[^ ^7^, ^8^, ^13^, ^14^ ^]^ the viability of cells and microbes in double emulsions,^[^ ^4^, ^15^, ^16^ ^]^ response under electric field,^[^ ^17^ ^]^ and the heat flux of microcapsules with phase change materials.^[^ ^18^, ^19^ ^]^

Among various methods for producing double emulsions,^[^ ^20^, ^21^ ^]^ microfluidic droplet generation stands out for its ability to produce monodisperse double emulsions with precisely controlled geometry and dimensions.^[^ ^22^, ^23^, ^24^ ^]^ Moreover, by completely keeping the inner and the outer phases separated during the formation of double emulsions, microfluidic generation ensures that encapsulation of materials in the core (inner) phase is performed with high efficiency. By adjusting generation conditions, the properties and the dimensions of double emulsion droplets can be modified continuously during production.^[^ ^25^, ^26^, ^27^, ^28^ ^]^ Our recent studies have also shown that double emulsion generation can be parallelized to produce large volumes of uniform droplets for practical applications.^[^ ^29^, ^30^ ^]^

To optimize double emulsions for a specific application, it is often necessary to prepare a large set of distinct double emulsion droplets with carefully controlled dimensions and compositions.^[^ ^14^, ^31^ ^]^ In a recent study, for example, over 80 different formulations had to be prepared and tested to find a double emulsion that could be used to prepare ultrasound responsive capsules.^[^ ^32^ ^]^ Preparing such a large number of double emulsions with distinct properties using microfluidics offers a powerful approach to facilitate screening and optimization of the dimension and the composition of double emulsions.^[^ ^33^, ^34^, ^35^ ^]^ Such droplet libraries accelerate the identification of optimum samples and can also provide large datasets that could be used for machine learning‐enabled discovery of new materials.

Generating high quality double emulsion droplet libraries, however, poses several challenges. The process requires time‐consuming and laborious operation of the droplet generator. Microfluidic generation of double droplets is susceptible to disruptions, including pump fluctuations, instrument biases, vibrations, and impurities, which are difficult to predict and control.^[^ ^36^, ^37^ ^]^ Consequently, continuous monitoring and intervention by a skilled operator are necessary to maintain the quality of droplet libraries generated using microfluidic devices.

With recent advances in artificial intelligence (AI) technologies and enhancements in computing powers, numerous studies have explored applying AI in microfluidic systems to automate tasks and augment human expertise.^[^ ^36^ ^]^ For example, AI has been used to automate the design of microfluidic droplet generators,^[^ ^38^ ^]^ to enable label‐free droplet detection and sorting,^[^ ^39^, ^40^ ^]^ and to perform autonomous flow chemistry experiment for quantum dot synthesis.^[^ ^41^ ^]^ In a previous study, our group enhanced in the consistency of microbubble generation using a deep learning‐based classification model and feedback control.^[^ ^37^ ^]^ Despite these advances, most studies to date have focused on using AI‐based techniques to facilitate relatively simple microfluidic systems such as single droplet generators. Double emulsion generation poses significantly greater challenges than single droplet generation because of the presence of three separate liquid phases. Double emulsion generation can be easily disturbed, leading to production of emulsions with heterogeneous structures due to jetting or satellite droplet formation. Moreover, the inner phase flow can deviate from the middle phase flow—a common failure mode in single‐junction double emulsion generators like glass capillary devices—, leading to severe loss of reagent and time, and even damage of the microfluidic device.^[^ ^42^ ^]^ This type of failure is particularly problematic because double emulsion generation cannot be easily recovered, and its occurrence is difficult to predict. These challenges have been major obstacles to the automation of double emulsion generation.

In this study, we demonstrate an automated droplet library generator (ADLib) by employing an AI‐empowered algorithm. This library generator automates critical stages of the droplet generation process, enhancing productivity and accelerating identification and discovery of double emulsions for specific applications. The system integrates a convolutional neural network (CNN)‐based object detection model, decision‐making and feedback control algorithms, and a graphical user interface (GUI). Using one of the latest object detection models, YOLOv10n, the system enables real‐time monitoring of droplet generation. Based on detection results, the program controls pumps and actuators to maintain double emulsion generation consistency, restoring the system from failure modes and collecting monodisperse products. Feedback control allows for the production of double emulsion droplets with user‐defined properties by adjusting the flow rates of the liquid phases. The GUI supports user interaction, facilitating the use of the ADLib. Together, the object detection model, GUI, algorithms, and hardware function as the operator's “eyes,” “brain,” and “hands,” enabling complete automation of double emulsion droplet library generation. We anticipate that the ADLib will accelerate high throughput screening and discovery across multiple fields including biomedicine, cell biology, and material synthesis.

## 2 Results and Discussion

### 2.1 Automated Droplet Library Generator (ADLib)

**Figure 1:**
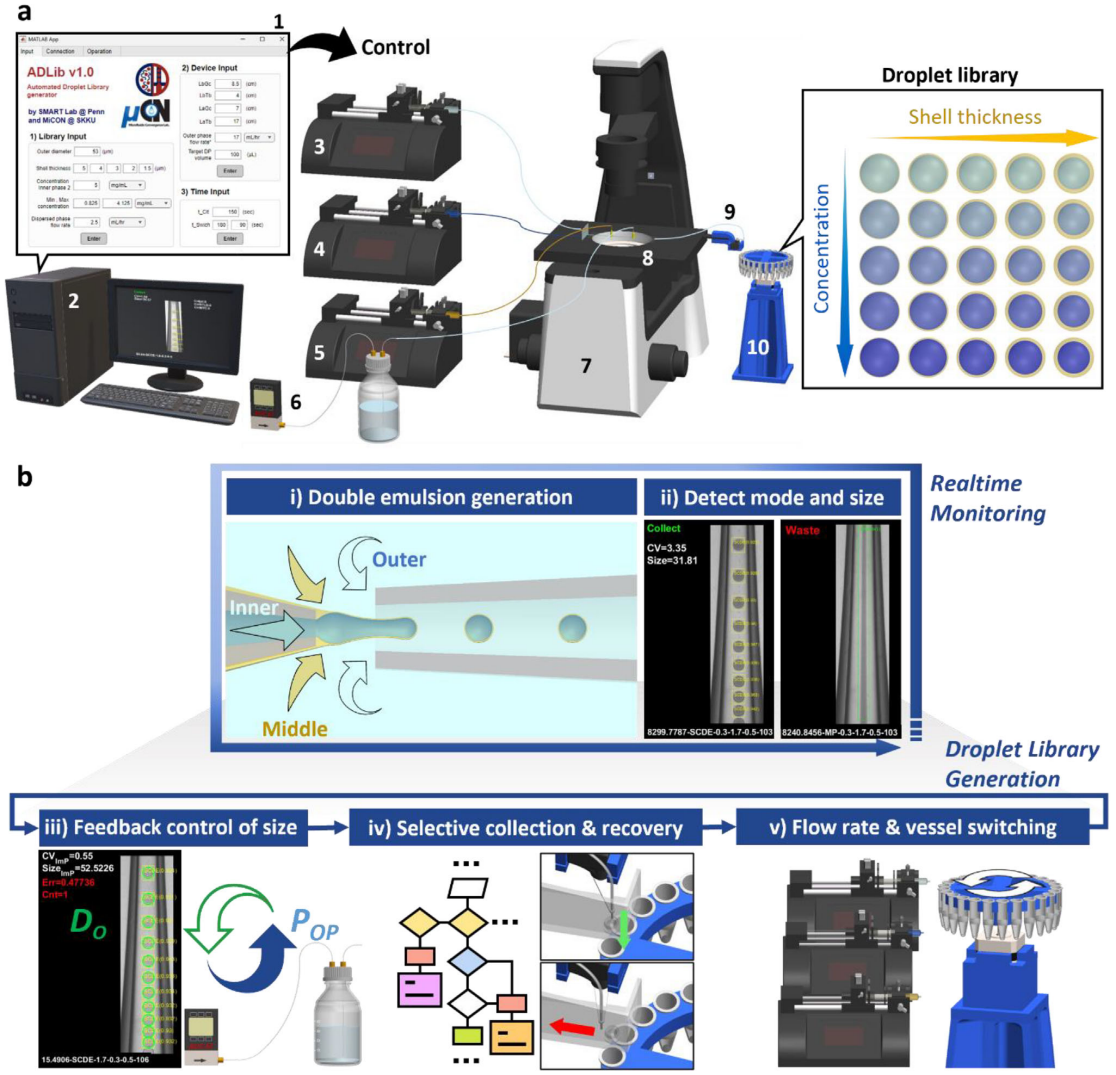
Automated droplet library generator (ADLib). a) Schematic illustration of ADLib, consisting of: (1 and 2) a graphical user interface (GUI) on a computer; (3–5) three syringe pumps for the inner phase 1 and 2, and the middle phase; (6) a pressure controller and reservoir for the outer phase; (7) a high‐speed camera installed on an optical microscope; (8) a microfluidic device incorporating a droplet generator and a microfluidic mixer chip; (9) a product selector; and (10) a rotational collector. b) Operation of the automated droplet library generator program: (i and ii) continuous real‐time monitoring of droplet generation images, and (iii–v) the three primary functions for the generation of a droplet library: (iii) regulation of droplet size by feedback control of the outer phase pressure, (iv) selective collection of single‐core double emulsion droplets by a decision‐making algorithm, and (v) adjustment of flow rates and vessel switching for generation and collection of distinct double emulsion droplets.

The automated droplet library generator (ADLib) consists of a glass capillary double emulsion droplet generator, a microfluidic mixer, three syringe pumps, a pressure controller, an inverted microscope equipped with a high‐speed camera, a handling system that includes a product selector and a rotational collector, as illustrated in **Figure** **1**a. The microfluidic mixer combines two inner‐phase solutions—aqueous solutions with and without an Evans Blue—to create a series of inner phases with different concentrations of the dye. A product selector then directs the resulting double emulsion droplets to either a collection vessel or a waste vessel. The rotational collector, which holds 25 micro tubes, positions each tube under the outlet tubing for sample collection. The two inner‐phase solutions and the middle‐phase solution are introduced into the glass capillary device using syringe pumps, while the outer‐phase solution is controlled by pressurizing a reservoir, enabling precise handling of a large liquid volume. These instruments are connected to a personal computer and are controlled by a custom‐developed droplet library generation program. This program features an object detection model fine‐tuned on a custom dataset, feedback control and decision‐making algorithms, and graphical user interface (GUI) for user interaction (Figure S1, Supporting Information).

The ADLib executes several operations to produce a double emulsion droplet library, including analyzing droplet generation mode, controlling the syringe pumps and the pressure controller, and managing the product selector and the rotational collector. The program analyzes droplet generation images in real‐time to detect droplet generation mode and droplet size, as shown in Figure 1b(i,ii). The program varies the flow rates of the fluid phases to adjust the droplet size to meet the target specification. Once single‐core double emulsion (SCDE) droplets with the specified dimensions are detected, the product selector moves the outlet tubing to collect the double emulsion as shown in Figure 1b(iii,iv). If a failure mode is detected (i.e., the device is not operating in the SCDE mode), the program promptly halts the collection of droplets and triggers the recovery of the SCDE generation mode (detailed description is provided in section 2.3). After collecting a specified amount of emulsions, the flow rate settings and collection vessels are adjusted to collect droplets with different dimensions or inner phase solution concentrations (Figure 1b(v)). By varying the flow rate ratio of the middle phase over the inner phase and altering that of the two inner phases, the ADLib produces 25 distinct types of double emulsions with different shell thicknesses and inner phase solute concentrations (Figure 1a).

### 2.2 Main Functions for the ADLib

#### 2.2.1 Droplet Generation Monitoring Using Object Detection Model

**Figure 2:**
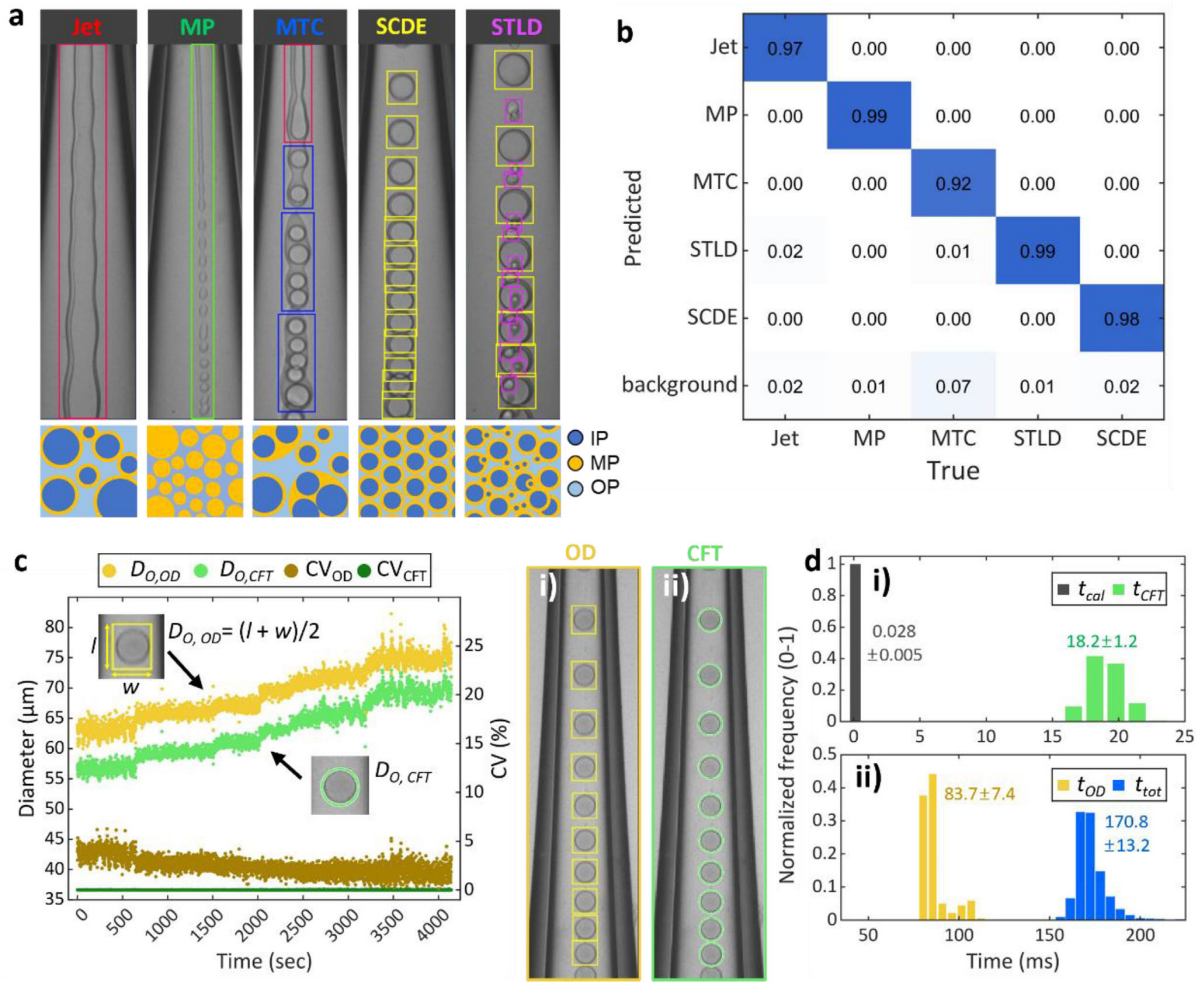
Fine‐tuning and performance evaluation of YOLOv10n object detection model. a) Classes of objects that are detected in different modes of double emulsion generation, along with examples of droplet generation images with annotations and illustrations of droplets produced in each generation mode. IP, MP, and OP represent the inner, middle, and outer phase fluids, respectively. The width of the droplet generation images is equivalent to 200 µm. b) Normalized confusion matrix of the fine‐tuned object detection model tested on the validation data. c) Comparison of the outer diameter (*D_O_*) and coefficient of variation (CV) of double emulsion droplets: i) approximated from the object detection (OD) results, and ii) determined using the circle fitting method (CFT). The width of the droplet generation images is equivalent to 256 µm. d) Histograms show the distribution of processing times for size calculation from detection results (*t_cal_*), OD (*t_OD_*), and CFT (*t_CFT_*), and the overall image processing time (*t_tot_*) for a single loop of droplet generation monitoring process. The numbers on the histogram are the mean value and standard deviation of the image processing times.

The object detection model is used to gather information, such as droplet generation mode and droplet size, from the high‐speed camera images of flows in the microfluidic device. Double emulsion generation is significantly more complex than single emulsion formation due to the flow of three liquid phases and the concurrent break‐up of the inner and middle phases, which leads to a wide variety of flow behavior.^[^ ^23^, ^25^, ^28^ ^]^ The types of double emulsion droplet generation modes in microfluidic devices vary across studies, and depend on the geometry of the double emulsion generator. In this work, to enable automated monitoring and control of droplet generation, we define five distinct droplet generation modes: jetting (Jet), middle phase only (MP), multicore droplets (MTC), single‐core double emulsion (SCDE), and satellite droplets (STLD) (**Figure** **2**a). Among these, SCDE is the desirable mode for most applications, producing monodisperse double emulsion droplets with single inner droplets encapsulated by middle‐phase droplets.^[^ ^21^ ^]^ The other modes are considered undesirable in this study, as they often result in the formation of non‐uniform double emulsions or single emulsion droplets without inner core droplets. For instance, the Jet mode occurs when the outer phase or dispersed phase flow rate surpasses critical values, resulting in an elongated compound jet of the middle phase and the inner phase, producing polydisperse droplets. When the middle and outer phase flow rates increase, droplet generation shifts to the MTC mode, generating multi‐core double emulsion droplets. A decrease in the outer phase flow rate yields satellite droplets along with SCDE droplets (the STLD mode). If the middle phase flow rate decreases, the inner phase can deviate from the middle phase and no double emulsions are generated (the MP mode). All these modes of double emulsion generation can also occur unexpectedly under the same flow conditions due to unknown disturbances, which is detrimental in producing highly uniform double emulsions during an extended period of operation (Video S1, Supporting Information).

To distinguish these modes, we use a convolutional neural network (CNN)‐based object detection model, YOLOv10n, which we fine‐tune using a custom data set. The dataset is constructed with 1351 images of droplet generation under varying flow conditions. At least 100 images representing each mode are included. To enhance the general applicability of the object detection model and avoid overfitting, the image data sets are obtained from experiments using three double emulsion droplet generators with different geometries (Figure S2, Supporting Information). Objects in the images are annotated with bounding boxes and labels as shown in Figure 2a. We classify and label objects into five classes (Jet, MP, MTC, SCDE, and STLD) corresponding to the five droplet generation modes. The Jet and MP modes generally contain a single object per image; thus, we include a large number (>890) of images for these two modes in the data set. We increase the total number of images used for fine‐tuning to 3513 by data augmentation, which increases the diversity of data set by adjusting the brightness, blur, and exposure of images. Fine‐tuning is completed in under an hour on a single graphical processing unit (GPU) workstation. The details of the fine‐tuning process are described in the experimental section.

Normalized confusion matrix, which is a metric that evaluates the classification performance of the fined‐tuned model, indicates that over 98% of validation images are correctly predicted for all classes as shown in Figure 2b. The mean average precision (mAP) score, a key metric for assessing object detection models, is calculated to evaluate both classification and localization performances, which indicate how accurately the model predicts the class and location of objects, respectively. The fine‐tuned model achieves a mAP of 0.9682 at an intersection over union (IoU) threshold of 50, and 0.7457 for IoU values ranging from 50 to 95, indicating high accuracy. Furthermore, the model accurately detects objects in experiments with different droplet generators of varying geometries and solutions with different viscosities and refractive indices, demonstrating robust generalization performance (Figures S3 and S4, Supporting Information).

We choose the object detection model over the classification model, which is a widely used computer vision method for microfluidics research.^[^ ^37^, ^39^, ^42^ ^]^ To compare the performance of these two CNN‐based models in correctly detecting different modes of double emulsion generation, we fine‐tune an InceptionV3 classification model using the same dataset, achieving a validation accuracy of 0.9656 (Table S1, Supporting Information). Despite its high accuracy, the classification model often misclassifies new images; for example, this model misclassifies an image with a small jet and several single core double emulsion droplets to be in the SCDE mode (Figure S5a, Supporting Information). This misclassification occurs likely because the new image predominantly features SCDE objects as shown in Figure S5b (Supporting Information). In contrast, the object detection model correctly identifies the short compound jet at the top of the image, classifying it correctly as the Jet mode (Figure S5c, Supporting Information).

Another key advantage of the object detection model is its ability to rapidly measure the size of SCDE droplets. In typical microfluidic experiments, droplet sizes are measured by fitting droplet images to circles and calculating their diameters, which requires an additional image processing procedure. To streamline this process, we test the localization performance of the object detection model in measuring the size of double emulsions. Bounding box data from object detection is used to approximate the diameters of SCDE droplets (*D_O, OD_*), as the average of length and width of the rectangular bounding box (*D_O, OD_* = (*l* + *w*)/2); these values are very similar to the size of droplets determined using the circle fitting method (*D_O, CFT_*) on MATLAB (Figure 2c). For example, regardless of the droplet size, *D_O, OD_* is consistently 6–8 µm larger than *D_O, CFT_*, which is equivalent to just 3–4 pixels. This performance demonstrates that the object detection model is proficient in determining the droplet size and can reliably monitor the trends in the size change. Minor variations in this size difference are influenced by droplet characteristics such as shell thickness and color.

This approximated size from object detection allows for a highly reliable collection of monodisperse SCDE droplets by comparing the current size to the previous size and verifying that the coefficient of variation (CV) of the size remains below the threshold value (Figure S6, Supporting Information). For instance, even if the object detection model misclassifies the other modes as the SCDE mode, the program can still reject polydisperse SCDE droplets by rapidly measuring the CV. Additionally, this approach streamlines image processing workflow. Processing times for calculating *D_O_* from detection results (*t_cal_*) and the circle fitting (*t_CFT_*) are ∼0.028 ms and ∼18.2 ms, respectively, on a personal computer with a single GPU (Figure 2d(i)). In the droplet generation monitoring loop, the total processing time per image (*t_tot_*) is ≈171 ms, which includes tasks such as loading high‐speed images, object detection (*t_OD_* ≈ 83.7 ms, inference time), visualizing detection results, decision‐making, and saving images (Figure 2d(ii); Figure S6, Supporting Information). Since *t_cal_* is negligible, eliminating circle fitting saves ≈11% of the total processing time, which is equivalent to the time elapsed for the generation of ≈160 double emulsion droplets. In short, the object detection model is extremely valuable for monitoring droplet generation, offering highly accurate determination of both droplet size and generation mode.

### 2.3 Selective Collection of SCDE Droplets and SCDE Generation Mode Recovery

To collect only SCDE droplets, the ADLib uses a decision‐making algorithm to control the position of the outlet tubing as illustrated in Figure S6 (Supporting Information). The outlet tubing is connected to the product selector which is driven by a servo motor to rapidly adjust the position of the outlet tubing (≈30 ms) to direct the undesired by‐products into a waste vessel; the motion of the product selector is performed while minimizing any fluctuations in droplet generation (Video S2, Supporting Information). Details about the decision‐making algorithm and the product selector operation are provided in the supporting information (Figure S6, Supporting Information).

**Figure 3:**
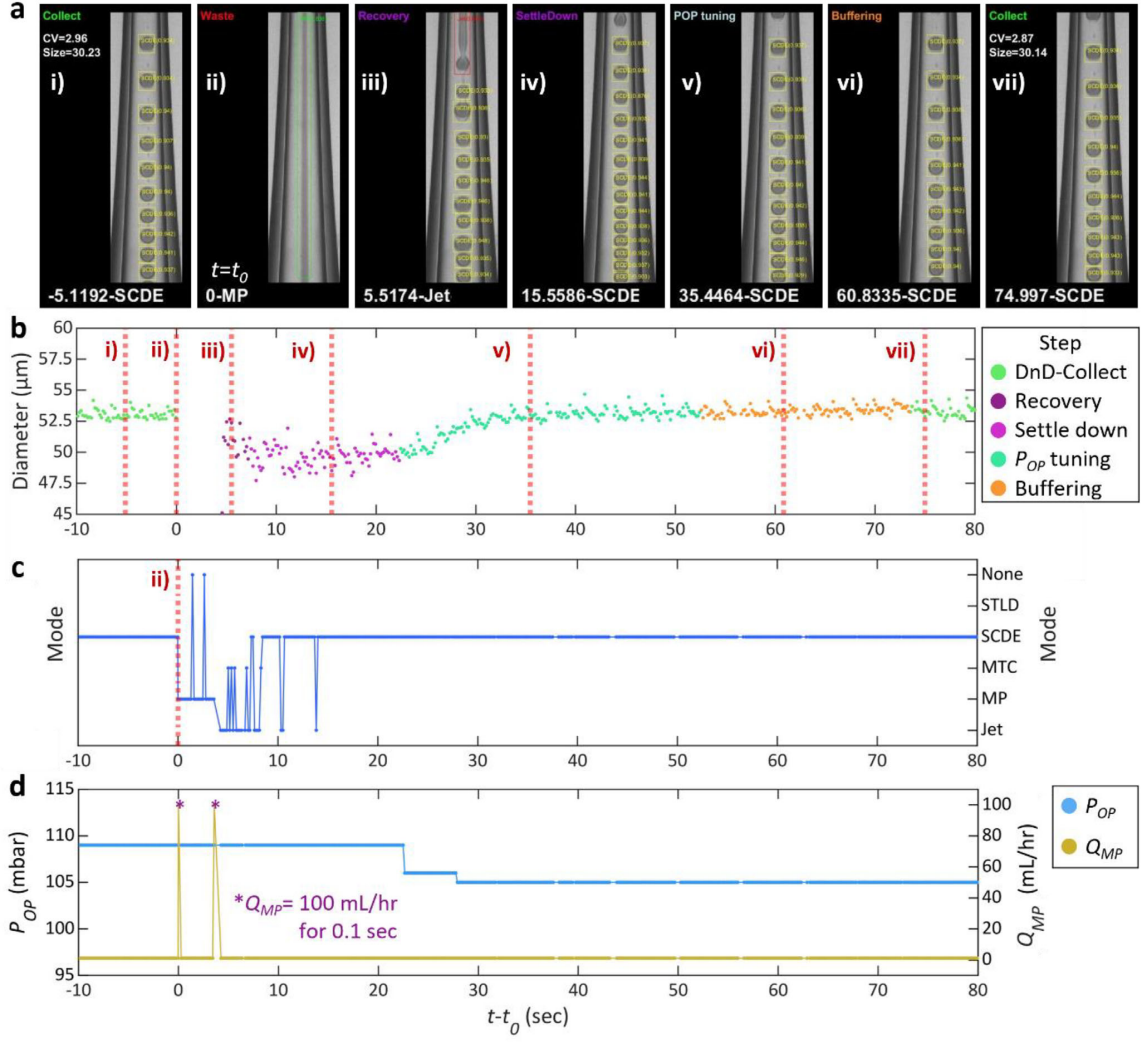
Single‐core double emulsion (SCDE) generation recovery process. a) Droplet generation images and detection results during the SCDE recovery process. Information on the top left and bottom left of each image displays the current step, time, and droplet generation mode, respectively. Flow rates for inner phase 1 and 2, and the middle phase are 1.7, 0.3, and 0.5 mL h^−1^, respectively. Droplet generation undergoes the following changes due to unknown disturbance and a recovery process that is executed by the droplet library generation program: i) the generation and collection of SCDE droplets, ii) the deviation of the inner phase from the middle phase and detection of MP mode (*t* = *t_0_*), iii) the restoration of the coaxial jet with the middle phase enveloping the inner phase by pulsing the middle phase flow, iv) the stabilization of the droplet generation system, leading to the generation of SCDE droplets, v) feedback control of *D_O_* by tuning *P_OP_*, vi) waiting for defects to flow out of the device, and vii) the resumption of SCDE droplets collection. The width of the droplet generation images is equivalent to 256 µm. b–d) Temporal changes in (b) the outer diameter of double emulsion droplet, (c) the detected droplet generation mode, (d) the outer phase pressure (*P_OP_*), and the middle phase flow rate (*Q_MP_*) during the SCDE recovery process.

During double emulsion generation, the microfluidic device that is operating in the SCDE mode can enter any one of the other four modes due to the changes in the flow rates or even without any changes in the flow rates due to unexpected disturbances, such as vibrations or impurities in the fluid. The microfluidic operation can return to the SCDE mode from the Jet, MTC, and STLD modes if the flow rates are restored, and disturbances are removed. However, if the inner phase deviates from the middle phase, resulting in the MP mode, recovery to SCDE mode is impossible without aggressive intervention (Video S1, Supporting Information). Furthermore, the MP mode can arise across a wide range of flow rate settings and in almost all glass capillary devices, regardless of their dimensions, even without apparent disturbances. Over the course of ten experiments using our ADLib system, the MP mode was observed 195 times during a total experimental duration of 26 h 16 min 17 sec (Table S2 and Figure S7, Supporting Information). Restoring the microfluidic operation from the MP mode back to the SCDE mode often relies on somewhat arbitrary adjustments by an experienced user. This process may involve manual adjustment of the flow rates and occasionally applying mechanical agitation to the tubing, syringe pump, or the device itself. We automate this recovery process (**Figure** **3**; Figure S6 and Video S3, Supporting Information).

Double emulsion generation can transition from the SCDE mode to the MP mode without any apparent cause as shown in Figure 3a–c, and Video S1 (Supporting Information). The library generation program detects this transition immediately, changes the position of the product selector to direct the by‐products into the waste vessel, and initiates the recovery process. Inspired by how experienced operators gently tap the tubing for the inner and middle phases to recover the SCDE mode in such a situation, the program applies a strong pulse to the middle phase flow: the flow rate briefly increases to 100 mL h^−1^ for 0.1 seconds then restores to its original value. This step is repeated until a co‐axial compound jet of the middle and the inner phases is recovered as shown in Figure 3a,b(iii) (“recovery” step). This particular example in Figure 3d required two pulses. The program then waits a specified amount of time (15 sec) for the droplet generation system to reach a steady state (“settle‐down” step) (Figure 3a,b(iv)). If the diameter of SCDE droplets differs from the size that was detected prior to the implementation of the recovery step, the program triggers a proportional (P) control over the pressure exerted on the outer phase (*P_OP_*) to control the droplet size (“*P_OP_* tuning” step) (Figure 3a,b(v),d). This step is essential, as the profile of the interface between the middle and the outer phases in the microfluidic channel can change after the recovery step, resulting in different SCDE droplet size even if there were no changes in the flow rates of the three phases (Figure S8, Supporting Information). The feedback control for droplet size will be discussed in detail in the next section. The program subsequently waits for off‐spec droplets to flow out of the device (“buffering” step) (Figure 3a,b(vi)) and, the library generator resumes the collection of SCDE droplets (Figure 3a,b(vii)).

#### 2.3.1 Droplet Size Regulation by Feedback Control

The dimensions of double emulsion droplets depend on the flow rates of the three fluid phases, the geometry of the microfluidic device, and the properties of the fluids such as viscosity, density, and interfacial tension.^[^ ^23^, ^25^, ^28^, ^43^ ^]^ Although relationships that correlate these parameters to the dimensions of double emulsion droplets have been reported, generating double emulsions with target dimensions often requires the involvement of operators with extensive knowledge and experience in microfluidic droplet generation. Automating control over double emulsion droplet dimensions is essential to streamline the preparation of a double emulsion library.

**Figure 4:**
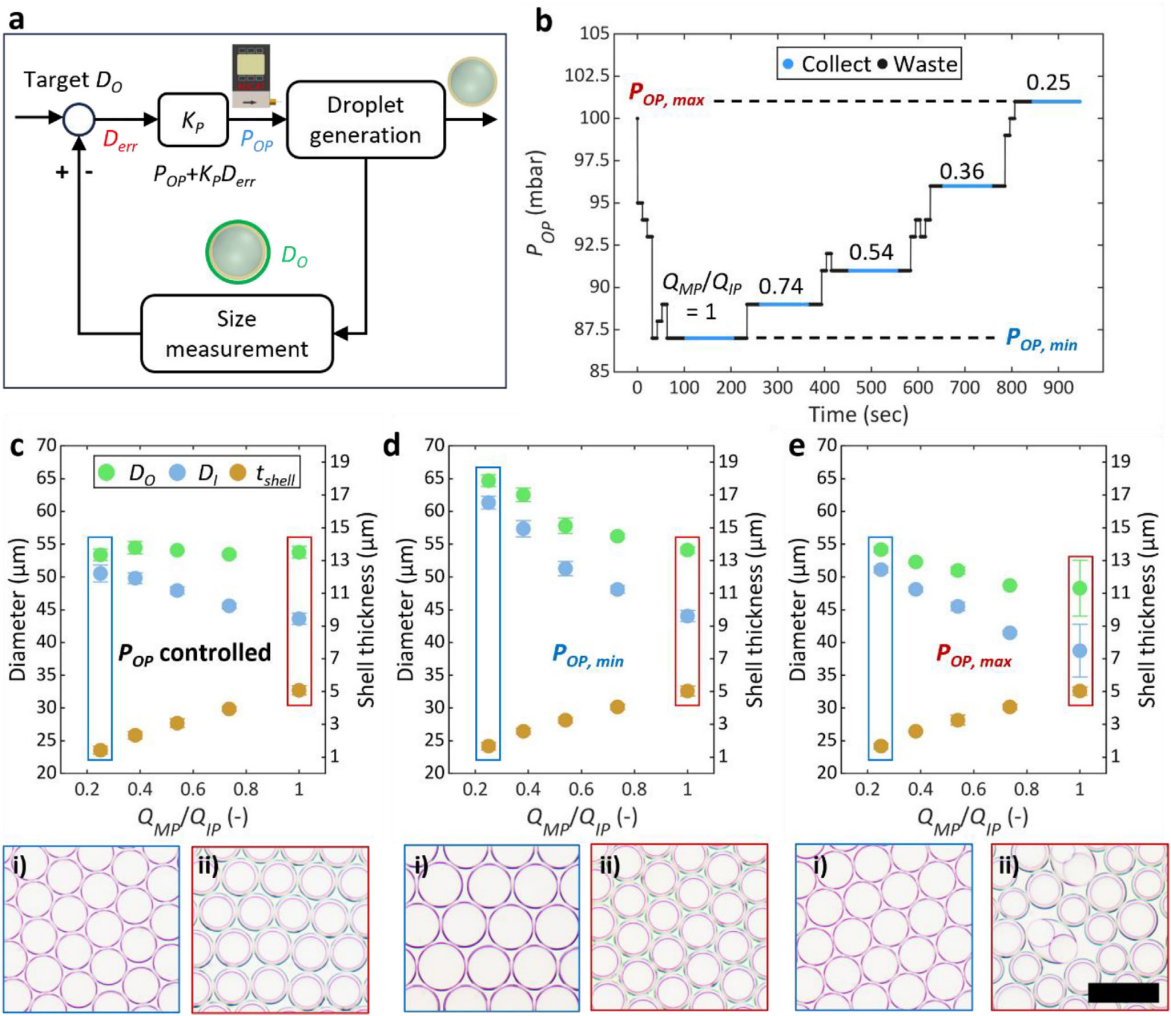
Feedback control of double emulsion droplet size by adjusting the outer phase pressure. a) Flowchart of the droplet size feedback loop. Arrows indicate flow directions. The program compares the current outer diameter (*D_O_*) of the droplet with the target diameter (target *D_O_*) and updates the outer phase pressure (*P_OP_*) via a proportional controller with the proportional gain, *K_P_*. The feedback loop continues updating *P_OP_* until the outer diameter error (*D_err_*) is <2% of the target *D_O_*. b) Temporal variation of *P_OP_* adjusted by the feedback control, with a dispersed flowrate (*Q_DP_* = *Q_IP_* + *Q_MP_*) of 2.5 mL h^−1^, and a target *D_O_* of 55 µm. c–e) Inner and outer diameters (*D_I_* and *D_O_*) and shell thickness (*t_shell_*) of double emulsion droplets as a function of the flow rate ratio of the middle phase to the inner phase (*Q_MP_*/*Q_IP_*) with (c) and without (d, e) feedback control. (i, ii) Optical micrographs of the double emulsion droplets produced under (i) *Q_MP_*/*Q_IP_* = 0.25 and (ii) *Q_MP_*/*Q_IP_* = 1. The scale bar is 100 µm.

To demonstrate the automated production of double emulsions using feedback control, we generate droplets with a constant outer diameter of 55 µm but varying in shell thickness as shown in **Figure** **4**. The outer diameter of double emulsions is controlled by changing the pressure exerted on the outer phase (*P_OP_*); the flow rates of the inner (*Q_IP_*) and middle (*Q_MP_*) phases are kept constant. The flow of the outer phase is pressure‐driven and thus responds faster to changes than the other phases which are driven by syringe pumps. We choose a P controller and determine its parameters using the Ziegler‐Nichols method as described in Supporting Information and Figure S9 (Supporting Information).

In the feedback control loop depicted in Figure 4a and Figure S10 (Supporting Information), the program measures the outer diameter of double emulsion droplets (*D_O_*) and compares it with the target diameter (Target *D_O_*) to calculate an error (*D_err_*). *P_OP_* is updated with the output of the proportional controller with the proportional gain, *K_p_*. The loop continues adjusting *P_OP_* until *D_err_* is less than 2% of the target value. Because the diameter of droplets fluctuates slightly even under steady‐state, *D_O_* is calculated by taking an average of 10 measurements using the circle‐fitting method. Additionally, as the response of the pressure controller can occasionally be delayed, the escape condition of the feedback loop is conservatively programmed; if the size error *D_err_* is less than 2% of the target *D_O_*, the program waits briefly (0.5 sec) and checks *D_err_* again. The feedback control loop terminates if this condition is satisfied more than twice. If the droplet generation mode deviates from SCDE, the feedback control algorithm restores the SCDE mode. As shown in Figure S10 (Supporting Information), if the Jet or MTC mode is detected, the program decreases *P_OP_* by 5 or 1%, respectively. If the STLD mode is detected, *P_OP_* is increased by 1%. These *P_OP_* adjustments follow the inverse process of the flow rate changes that trigger the transition from the SCDE to other modes. Such pressure adjustments are continuously made until the SCDE mode is restored. If the MP mode is detected, the program initiates the recovery process as described in section 2.3.

As a result of the feedback control, the outer phase pressure is adjusted to meet the target diameter of 55 µm; the shell thickness is varied by changing the ratio of the middle and inner phase flow rates (*Q_MP_*/*Q_IP_*) (Figure 4b). Double emulsion droplets produced with feedback control have a constant outer diameter regardless of the variations in *Q_MP_*/*Q_IP_* (Figure 4c). In contrast, without feedback control, the outer diameter fluctuates significantly with changes in *Q_MP_*/*Q_IP_* (Figure 4d,e). More detrimentally, droplet generation often shifts out of the SCDE mode (Figure 4e). The size and morphology of double emulsion droplets produced under constant outer phase pressure vary significantly as *Q_MP_*/*Q_IP_* changes (Figure 4d,e(i,ii)). Here, the double emulsions are collected regardless of their size and morphology to clearly observe the importance of the feedback control. This variation in *D_O_* and droplet generation mode is likely due to the changes in the outer phase Capillary number—a dimensionless quantity representing the relative influence of viscous drag force to interfacial tension force and significantly impacting droplet break‐up. The dependence of changes in *D_O_* by *Q_MP_*/*Q_IP_* on the presence of a surfactant in the middle phase suggests the following: as the middle phase layer becomes thinner (i.e., as *Q_MP_*/*Q_IP_* decreases), the local surfactant coverage at the interface diminishes. This reduction increases interfacial tension and decreases the Capillary number, leading to the generation of larger droplets at a lower frequency (Figure S11, Supporting Information). These results underscore the importance of feedback control for droplet library generation. Feedback control enables the generation of droplets of specified dimensions under a range of conditions, without requiring in‐depth knowledge or expertise in droplet microfluidics.

### 2.4 Double Emulsion Droplet Library Generation

**Figure 5:**
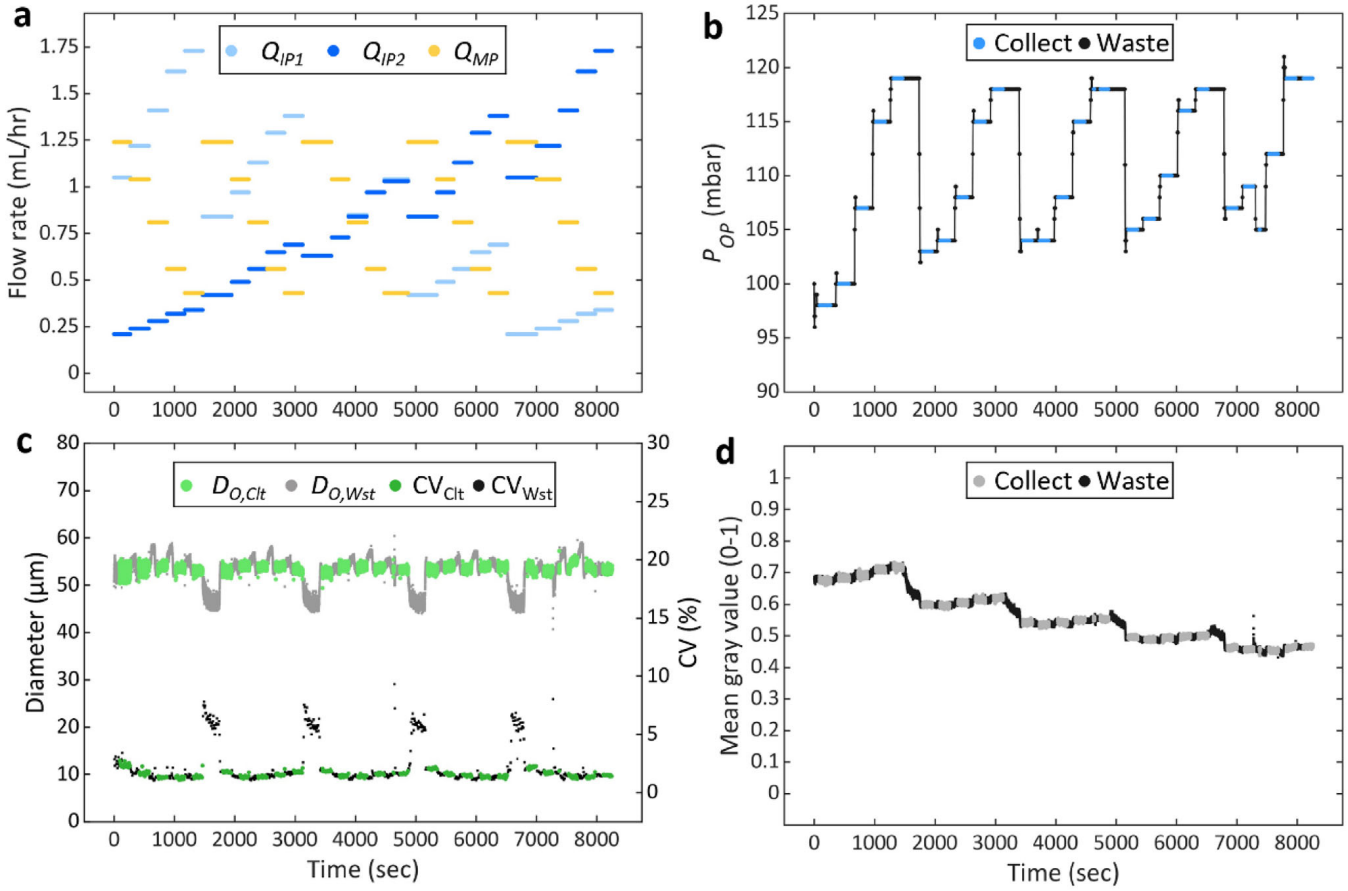
Generation of a double emulsion droplet library with varying solute concentration and shell thickness. a) Flow rates for inner phase 1 and 2, and the middle phase, used to generate a 5 × 5 double emulsion droplet library. The flow rates are calculated by the droplet library generation program based on the following inputs: target droplet size = 53 µm, shell thicknesses = [5, 4, 3, 2, 1.5] µm, solute concentration of inner phase 2 = 5 mg mL^−1^, minimum and maximum solute concentration in the inner phase = [0.825, 4.125] mg mL^−1^, and dispersed phase flowrate = 2.5 mL h^−1^. b) Temporal variation of the outer phase pressure as adjusted by feedback control. c) Outer diameter (*D_O_*) and coefficient of variation (CV) of double emulsion droplet as functions of time. d) Changes in normalized mean gray value of the double emulsion droplet in high‐speed images over time. The term “Collect” and the subscript “Clt” represent data from images when droplets are collected, while “Waste” and the subscript “Wst” refer to data from images of droplets flowing into the waste vessel.

To demonstrate the versatility of the ADLib, we produce a 5 × 5 double emulsion droplet library with constant *D_O_* and varying shell thickness and solute concentration in the inner phase. As presented in Figure S1 and Videos S4 and S5 (Supporting Information), the ADLib GUI allows researchers to input parameters to generate 25 different types of double emulsion droplets with five distinct shell thicknesses and inner phase concentrations. The program calculates a 25 × 3 flow rate matrix for inner phase 1 and 2, and the middle phase, using the input values such as the target outer diameter, shell thicknesses, solute concentration of the inner phase 2, the concentration range of the solute in the core of double emulsions and a dispersed phase flowrate (*Q_DP_* = *Q_IP1_* + *Q_IP2_* + *Q_MP_*) (Figure S12, Supporting Information). The ADLib subsequently produces double emulsion droplets by varying the flow rates of the inner and middle phases following the flow rate matrix (**Figure** **5**a). If the MP mode is detected at the beginning of the experiment, the program recovers to the SCDE mode and then proceeds by implementing the size regulation and selective collection steps (Video S5, Supporting Information).

In the production of the double emulsion library, it is important to minimize the loss of reagent by reducing the time between sample collections. To achieve this goal, we organize the 25 flow rate settings into five subsets with each subset containing five distinct flow rate settings. Within each subset, *Q_MP_*/*Q_IP_* varies in five steps, which produces droplets with different shell thicknesses. At the end of each sub‐set, *Q_IP2_*/*Q_IP1_* is increased to change the concentration of the solute in the double emulsion. Prior to initiating the selective collection of the desired product under each flow rate setting, *P_OP_* is adjusted via feedback control to produce double emulsions with the target *D_O_* (Figure 5b). As a result, the outer diameter of the collected SCDE droplets remains at the target size of 53 µm with low CV, regardless of the flow rate ratio of the dispersed phases (Figure 5c). When the flow rate ratio changes, the selective collection pauses for specified period of time—90 sec after a change in *Q_MP_*/*Q_IP_* and 270 sec after a change in *Q_IP2_*/*Q_IP1_*—to allow the flows to be fully developed and reach a steady state within the microfluidic device (Table S3 and Figure S13, Supporting Information). This flow rate change time can be shortened to 30 sec when the single inner phase is used (Figure S14, Supporting Information). Changes in the inner phase solute concentration are verified by measuring the gray value of the inner phase in the droplet images. As displayed in Figure 5d, the mean gray value of the droplet remains essentially constant under each flow rate setting, indicating that the solute concentration of the inner phase is the same as calculated as the flow rate ratio of *Q_IP1_* and *Q_IP2_*, and the robustness of the library generation procedure. The gray value decreases in steps as solute concentration increases at the end of each sub‐set. A slight increase in gray value within each sub‐stage reflects a reduced core volume (and thus the inner diameter) in the double emulsion droplets.

**Figure 6:**
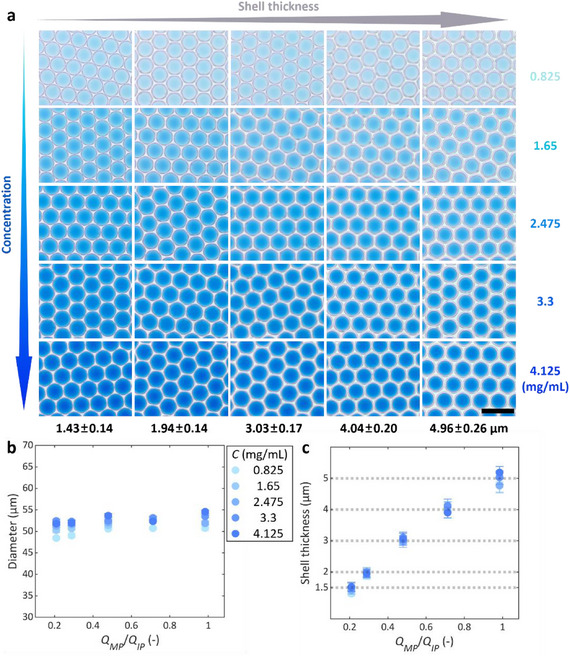
Double emulsion droplet library with varying concentration and shell thickness. a) Optical micrographs of double emulsion droplets from a 5 × 5 droplet library varying in the dye concentration and the shell thickness. The scale bar represents 100 µm. b) Outer diameter and c) shell thickness of the double emulsion droplets in the droplet library as a function of the flow rate ratio of the middle phase and the inner phase. “*C*” from the legend indicates the solute concentration in the inner phase of the double emulsion droplet.

Based on the strategy outlined above, the ADLib produces a 5 × 5 double emulsion droplet library automatically (Video S6, Supporting Information). The library generator successfully produces 25 distinct monodispersed SCDE droplets as shown in **Figure** **6**a. Along with the column direction in the image matrix, the inner phase solute concentration increases stepwise with the same increment from 0.825 to 4.125 mg mL^−1^. The five concentrations can also be specified by the user (Figure S1 and Video S5, Supporting Information). Double emulsion droplets in each image have a uniform color intensity within the inner droplets, indicating uniform solute concentration. From left to right of each row of Figure 6, the thickness of the shell increases (i.e., the size of the inner droplet decreases), resulting in a lighter blue intensity of the inner phase droplets with the same solute concentration. The outer diameters of the droplets are highly uniform at the target *D_O_* with CV less than 2.3% (Figure 6b). Given the dispersed phase flow rate of *Q_DP_* = 2.5 mL h^−1^ and the outer droplet diameter of *D_O_* = 53 µm, double emulsion droplets are generated at a frequency of ≈8.9 kHz. Although this frequency is significantly higher than the total image processing time (*t_tot_* ≈ 171 ms) of the ADLib program, no defect droplets are observed across all selectively collected samples, demonstrating the high reliability of the automated process by ADLib. Minor discrepancies in *D_O_* are due to the solute‐induced osmotic annealing of the double emulsions upon collection; that is, Evan's Blue, acting as a penta‐ionic electrolyte, increases the osmotic pressure of the inner phase and induces the diffusion of water from the outer phase into the inner droplets, increasing *D_O_* and *D_I_* while decreasing slightly *t_shell_*. The resolution limit of the high‐speed camera set‐up (2 µm/pixel) also likely contributes to the slight variations in *D_O_*. The shell thickness of the droplets closely matches the input values with an average error of 0.15 µm (Figure 6c), demonstrating that the device can precisely control droplet geometry according to user specifications. These results clearly demonstrate that the ADLib can reliably produce a double emulsion droplet library with user‐customized properties, which can significantly improve the speed and efficiency of optimization for droplet‐based materials.

## 3 Conclusion

In this study, we present an AI‐empowered automated double emulsion droplet library generator (ADLib) that incorporates a fine‐tuned CNN‐based object detection model, decision‐making and feedback control algorithms, and GUI. By analyzing the images of a microfluidic device under operation, the algorithms control hardware to adjust the flow parameters to ensure the generation and collection of double emulsions with single‐core droplets. The ADLib autonomously addresses unexpected disturbances that impede single‐core double emulsion formation without human intervention. The feedback control of outer phase pressure allows for the generation of droplets with the target outer diameter, regardless of shell thickness and solute concentration. The GUI enhances user accessibility, making the ADLib easy to use for individuals with limited programming or microfluidics experience.

Most of the components used to build the ADLib are equipment commonly used in droplet generation experiments. Key devices, such as microscopes, high‐speed cameras, syringe pumps, and pressure controllers, are compatible with ADLib regardless of their brand and models if they can communicate with a computer. The product selector and the rotational collector are made using 3D‐printed parts, hobby‐grade motors, and an Arduino; total cost is about USD 100. This handling system is highly cost‐effective, but the development of more advanced hardware would significantly enhance utility of the ADLib system. For example, developing a 96‐microwell plate‐compatible collector would expand the capacity of the ADLib and streamline droplet library analysis, making it compatible with a wide range of microwell‐based instruments.

Additionally, the dataset we are constructing and utilizing has been uploaded as “Glass capillary DE” to Roboflow and is publicly accessible. A personal computer with a mid‐spec GPU is sufficient to run the droplet library generation program. Owing to the robust generalization performance of the fine‐tuned objective detection model, the ADLib is applicable to glass capillary double emulsion generators with different geometries and dimensions. We are actively employing this approach in several ongoing studies. Expanding ADLib's application to droplet generators with significantly different geometries and materials, such as polydimethylsiloxane (PDMS)‐based double emulsion generators, would require redefining object classes, modifying datasets, and tuning algorithms to accommodate the new system configurations.

This AI‐empowered automation system for microfluidic experiments can automate most tasks traditionally performed by humans, with even greater efficiency. We are eager to share our ADLib with interested researchers and welcome their feedback to further enhance its functionality and user accessibility. In this study, we define five distinct droplet generation modes — SCDE as the desirable mode and the other modes (Jet, MP, MTC, and STLD) as undesirable modes — to automate double emulsion generation. However, various microdroplet morphologies with unique and useful properties can be produced under different droplet generation modes using specialized droplet generators.^[^ ^44^ ^]^ For example, Janus droplets,^[^ ^30^ ^]^ multicore double emulsion with controlled core numbers,^[^ ^45^, ^46^, ^47^ ^]^ triple and quadrable emulsions,^[^ ^48^ ^]^ and other complex droplets have been reported.^[^ ^49^ ^]^ Additionally, numerous failure and edge cases, such as channel wall wetting by the dispersed phase and nozzle clogging, present further challenges in droplet generator operation beyond the MP mode, which was the primary focus of this study. This highlights the significant potential for further automation in this field. We believe that ADLib can be extended to automate a wide range of droplet generation modes that have been achieved manually by a human operator. We hope that the ADLib will be widely adopted, freeing researchers from the tedious tasks involved in droplet generation experiments. The ADLib, we believe, will accelerate the optimization of double emulsions for various applications including cell encapsulation, drug delivery, and high throughput screening.

## 4 Experimental Section

### Experimental Setup

The automated droplet library generator consists of a personal computer (OptiPlex Tower Plus 7010, Dell), three syringe pumps (PHD ULTRA, Harvard Apparatus) corresponding to the inner phases 1 and 2 (IP1 and IP2), and the middle phase (MP), respectively, a presser controller (PCD Series, max pressure = 50 PSI, ALICAT scientific) for driving the outer phase (OP), a droplet generator, a micro mixer, a high‐speed camera (Fastcam mini AX200, Photron) attached to an optical microscope (ECLIPSE TE200, Nikon), a product selector, and a rotational collector (Figure 1a). The syringe pumps and pressure controller are directly connected to the computer by serial communication port. A high‐speed servo motor (S185, JR) and a stepper motor (NEMA‐17, Adafruit) were utilized as the moving parts of the product selector and the rotational collector, respectively. The remaining components of the product selector and collector (blue parts in Figure 1a) were fabricated using a fused filament fabrication (FFF) type 3D printer (M3‐ID, Makergear). The product selector and the collector are controlled by the computer through Arduino (Uno, Arduino). Droplet generation images were captured at 200,000 fps of shutter speed with a 512 × 1024 of original frame size and a 10× objective lens. The image resolution is 2 µm/pixel. Collected double emulsion droplets were further imaged with a CMOS camera (MU1400B, AmScope) attached to another optical microscope (Axioplan 2, Zeiss), achieving a higher image resolution of 0.7 µm/pixel for precise measurement of droplet dimensions. The optical micrographs in Figures 4 and 6 were obtained from this imaging setup.

### Droplet Generation

The droplet generator was fabricated using glass capillaries following previously reported methods.^[^ ^50^ ^]^ The inner and outer diameters of the injection nozzle are 26 and 64 µm, respectively, while those of the collection nozzle are 86 and 146 µm, respectively. The distance between two nozzles is 103 µm. Novec HFE‐7500 (3M) with 2% (v/v) perfluorinated lubricant (Krytox 157 FSH, Dupont) as a surfactant is used as MP. IP1 and OP are a 2% (w/v) PVA (MW 13k – 23k, Sigma Aldrich) aqueous solution, while 5 mg mL^−1^ of Evan's blue (Sigma Aldrich) was added to the PVA solution for IP2 to visualize the concentration difference of the inner phase. The two inner phase solutions were thoroughly mixed before injection into the droplet generator via a passive herringbone type mixer chip (Fluidic 1460 Chip, 400 µm width, Microfluidic ChipShop).

### Object Detection

The YOLOv10n object detector was fine‐tuned using a custom dataset. The droplet generation images were collected from experiments using droplet generators with three different geometries (Figure S2, Supporting Information). The images were cropped and then resized to a size of 128 × 640 pixel by padding black boxes. Objects in the images were manually annotated by drawing bounding boxes around each object and labeling them into one of five classes: A compound jet, a single jet of middle phase fluid, multicore double emulsion droplet, single‐core double emulsion droplet, and satellite droplet objects were labeled as Jet, MP, MTC, SCDE, and STLD, respectively. In this study, the dataset is constructed using an iterative refinement approach, incorporating additional failure images observed during early ADLib prototype testing. For each update, the study added images containing ≈100 objects in total, which significantly enhanced detection robustness. The final version of dataset consisted of 1, 353 images with 2, 252 SCDE, 1, artificial intelligence094 STLD, 766 MP, 621 MTC, and 335 Jet objects. The dataset was split into training and validation sets in an 8:2 ratio. Random variations in brightness, exposure, and blur were applied as augmentation, resulting in a total of 3513 images. The dataset was converted into YOLOv8 format. The annotation, argumentation, and dataset formatting processes were implemented using the Roboflow web application. The fine‐tuning was conducted using Python 3.90 and Pytorch 2.0.1 + cu118 on a workstation equipped with a single GPU (GeForce RTX 4090, NVIDIA) configured with CUDA Toolkit 11.8. and cuDNN 8.1.0. The batch size and the image size of the neural network were set to 64, and 640 × 640 pixels, respectively. The fine‐tuning process was completed in 54 minutes and 21 seconds, being terminated at epoch 259 due to early stopping. The best model, identified at epoch 159, was converted into MATLAB‐compatible ONNX (Open Neural Network Exchange) format without any performance drops and utilized for monitoring droplet generation. The converted model was run by MATLAB (2023b, MathWorks) on the personal computer equipped with a single GPU (GeForce RTX 3070, NVIDA).

### Droplet Generation Mode Classification From Object Detection Result

The object detection model outputs bounding box coordinates, class labels, and confidence scores for detected objects. Results are filtered by confidence thresholds of 0.5, 0.35, 0.75, 0.6, and 0.6 for the Jet, MP, MTC, SCDE, and STLD modes, respectively. The image is classified as the SCDE mode only if all detected objects belong to this class; if another class is present, it determines the droplet generation mode. When multiple non‐SCDE classes are detected, the mode is determined by a priority‐based decision‐making algorithm (Figure S6, Supporting Information). In rare cases (0.004%), when no objects are detected, the mode is classified as None.

## Conflict of Interest

I (Daeyeon Lee) have equity in InfiniFluidics.

## Supporting information



Supporting Information

Supplemental Video 1

Supplemental Video 2

Supplemental Video 3

Supplemental Video 4

Supplemental Video 5

Supplemental Video 6

## Data Availability

The data that support the findings of this study are available from the corresponding author upon reasonable request.
