# Supplementary material for: Artificial Intelligence‐Empowered Automated Double Emulsion Droplet Library Generation
Source: Small. 2025 Mar 25;21(18):2412099. doi: 10.1002/smll.202412099 (PMC12051774; doi:10.1002/smll.202412099)
Supplement: Supplementary file 1 — Supporting Information [file SMLL-21-2412099-s001.docx]

Supporting Information

Artificial Intelligence-Empowered Automated Double Emulsion Droplet Library Generation

Seonghun Shin, Owen D. Land, Warren D. Seider. Jinkee Lee*, and Daeyeon Lee*

S. Shin, O. D. Land, Prof. W. D. Seider and Prof. D. Lee

Department of Chemical and Biomolecular Engineering, School of Engineering and Applied Science, University of Pennsylvania, Philadelphia, Pennsylvania 19104, United States

E-mail: daeyeon@seas.upenn.edu

S. Shin and Prof. J. Lee

School of Mechanical Engineering, Sungkyunkwan University, Suwon, 16419, Republic of Korea

E-mail: lee.jinkee@skku.edu

Prof. J. Lee

Institute of Quantum Biophysics, Sungkyunkwan University, Suwon, 16419, Republic of Korea

Keywords: experiment automation, object detection, feedback control, microfluidics, convolutional neural network

**Contents**

**1. Graphical user interface of the automated droplet library generator (ADLib)**

**Figure S1.** Graphical user interface of ADLib.

**2. Fine-tuning of YOLOv10n object detection model**

**Figure S2.** Types of droplet generation images used for the construction of the dataset.

**Figure S3.** Object detection of droplet generation under various conditions.

**Figure S4.** Object detection of droplet generation with various solution combinations.

**3. Comparison of computer vision methods: classification and object detection**

**Table S1.** Comparison of training results of the YOLOv10n object detection model and the InceptionV3 classification model.

**Figure S5.** Comparison of prediction results of the YOLOv10n object detection model and the InceptionV3 classification model.

**4. Decision-making algorithm for selective collection and droplet generation mode recovery**

**Figure S6.** Flowchart of decision-making algorithm for selective collection of single-core double emulsions.

**Table S2.** Statistics of middle phase only (MP) mode occurrence in automated 5 × 5 double emulsion droplet generation experiments.

**Figure S7.** Temporal frequency of middle phase only (MP) mode occurrence in automated 5 × 5 double emulsion droplet generation experiments.

**Figure S8.** Single-core double emulsion generation recovery process without *P_OP_* tuning step.

**5. Droplet size regulation by feedback control**

**Figure S9.** Response of the outer diameter of double emulsion droplets (*D_O_*) to changes in the outer phase pressure (*P_OP_*).

**Figure S10.** Flowchart of the feedback control algorithm for adjusting double emulsion droplet size and droplet generation mode.

**Figure S11.** Effect of the flow rate ratio of the middle phase to the inner phase and the presence of surfactant on the outer diameter of the double emulsion droplet.

**6. Calculation of flow rates using user input**

**Figure S12.** Generation of flow rate matrix based on the user input parameters.

**7. Response of the double emulsion generator to flow rate changes**

**Table S3.** Effect of syringe type on the double emulsion generator response to the inner phase flow changes.

**Figure S13.** Response of the double emulsion generator to the inner phase flow rate changes based on syringe type.

**Figure S14.** Automated double emulsion droplet generation with a single inner phase solution and a flow rate changing time of 30 seconds.

**8. Supporting videos**

**Supporting video 1.** Failure of double emulsion generation.

**Supporting video 2.** Effect of product selector movement in droplet generation.

**Supporting video 3.** Single-core double emulsion generation recovery process.

**Supporting video 4.** Demonstration of graphical user interface 1.

**Supporting video 5.** Demonstration of graphical user interface 2.

**Supporting video 6.** Automated generation of double emulsion droplet library.

**1. Graphical user interface of the automated droplet library generator (ADLib)**

**
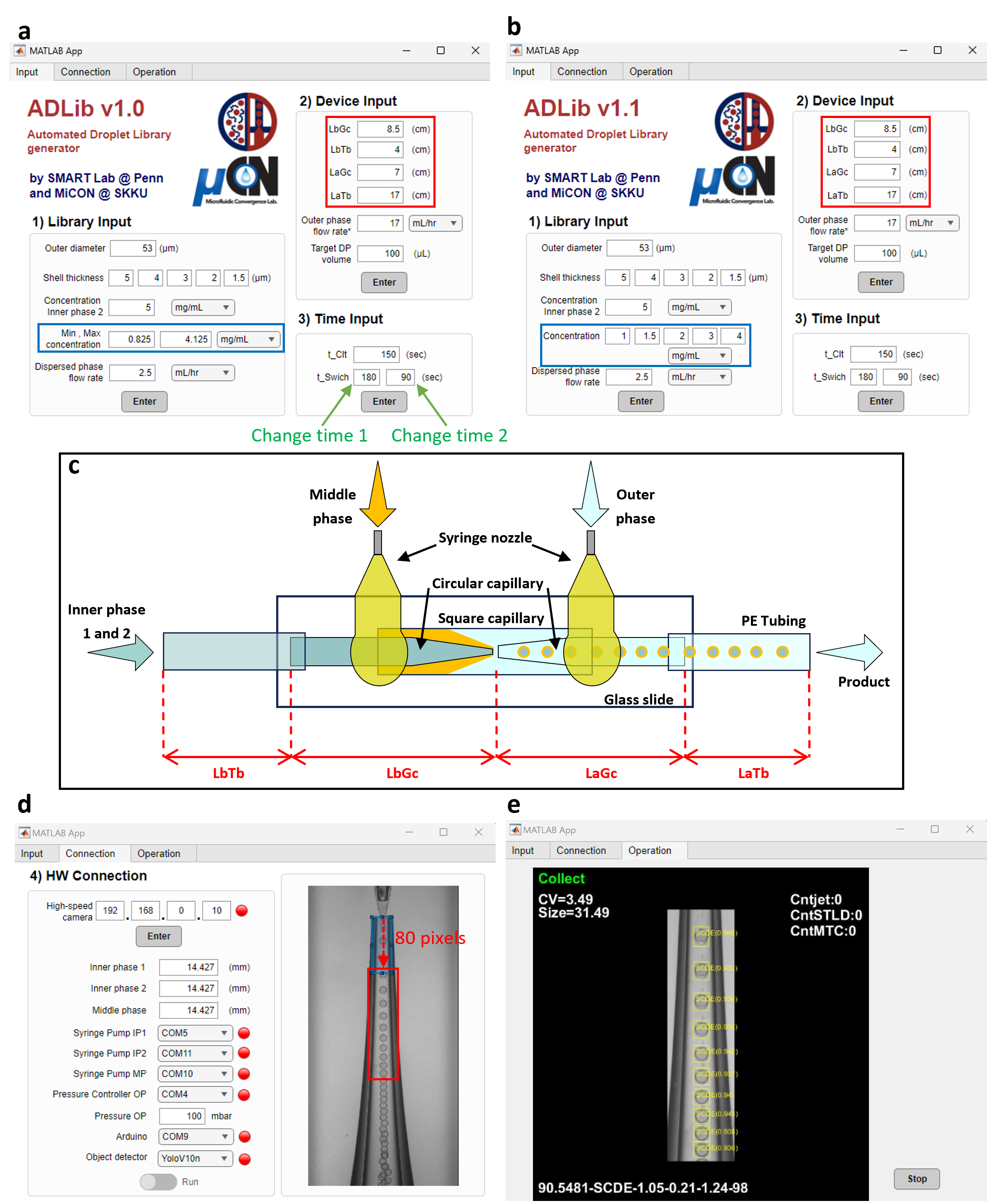
**

**Figure S1.** Graphical user interface (GUI) of ADLib. (a and b) GUI configuration of input tabs version (a) 1.0 and (b) 1.1. (c) Schematic illustration of glass capillary double emulsion generator, including descriptions of device inputs. d) HW (hardware) Connection tab, and e) Operation tab of the GUI.

The demonstration of the graphical user interface (GUI) program in **Video S4** and S5 aids understanding the description below.

**1) Library input**: The GUI program receives input values for calculating the flow rate matrix of inner phase 1 and 2, and the middle phase. “Outer diameter” is the target outer diameter of the double emulsion droplets. “Shell thickness” represents the thickness of the middle phase enveloping the inner phase in a double emulsion droplet. The program requires five shell thicknesses to generate a flow rate matrix for 5 × 5 droplet library generation. “Concentration Inner phase 2” specifies the solute concentration of the inner phase 2. “Min, Max concentration” of the input tab version 1.0 indicates the minimum and maximum solute concentration in the inner phase for the droplet library. The program calculates five linearly spaced concentration values between these two values. Alternatively, in the input tab version 1.1, researchers can directly input five target concentrations in the “Concentration” fields. “Dispersed phase flow rate” is the sum of the flow rate of the inner and middle phases. Once all input fields are submitted, the GUI generates and displays a 25 × 3 matrix of the flow rates for inner phase 1 and 2, and the middle phase.

**2) Device input**: The program calculates appropriate time intervals for steps based on the microfluidic device geometry. “LbGc”, “LbTb”, “LaGc”, and “LaTb” stand for the lengths of glass capillary (GC) and tubing (Tb) before and after the droplet generation junction, respectively as shown at Figure S1c. “Outer phase flow rate” is the estimated outer phase flow rate. Since the outer phase is pressure-driven, an exact flow rate measurement is not possible; instead, the minimum outer phase flow rate needed to generate single-core double emulsion droplets is used, based on prior experiments where the outer phase was injected via syringe pump. “Target DP Volume” represents the volume of each distinct type of double emulsion droplet that is to be collected. Once the user submits all fields, the GUI displays appropriate time interval: t_Ent_, t_Ext_, and t_Tub_. Here, t_Ent_ stands for the time for the inner phase to reach the droplet generation junction from the outlet of the micro mixer. It is calculated using the first inner phase flow rate from the flow rate matrix. Compared to t_Ent_, the time for the two inner phase flows to mix and depart from the micromixer is negligible. t_Ext_ represents the time for droplets, departing from the junction, to exit the microfluidic device. t_Tub_ is the time required to fill a collection vessel, e.g. microtubes, with the targeted dispersed phase volume.

**3) Time input**: “t_Clt” is the collection time for SCDE droplets of each type. The two fields next to “t_Switch” specify Change Time 1 and 2, which are time intervals for pausing collection to ensure complete changes in the flow rate ratio. When the flow rate ratio of the middle phase over the inner phase changes, the program pauses product collection for time specified by the Change time 2. When the flow rate ratio of the two inner phases changes, the collection halts for the sum of Change time 1 and 2. In this study, these intervals are determined empirically as shown in Table S3 and Figure S13.

**4) HW (hardware) connection**: The input fields, from top to bottom, correspond to the internet protocol (IP) address of the high-speed camera, the inner diameters of syringes for inner phase1 and 2, and the middle phase, and the initial outer phase pressure value. The drop-down menus enable users to select serial communication (COM) ports for connecting the devices and the type of object detection model; in this study, only YOLOv10n is exclusively used due to its superb performance. The color indicators on the right side of each row display the connection status of each device. When the toggle is set to “Run”, the program connects to the devices and displays the field of view from the high-speed camera that is installed on the microscope. The region of interest (ROI, red) for droplet generation image capture is defined as a rectangle with dimensions of 128 × 480 pixels, located 80 pixels from the top center of the user-drawn rectangle (blue)

**5) Operation**: The program displays the current droplet generation image with object detection results overlaid. On the image, the top-left corner shows, from top to bottom, the current step, the coefficient of variation (CV) and the average of the approximated droplet size (radius in µm). In the bottom-left corner, hyphenated text provides the elapsed time, detected droplet generation mode, flow rates for inner phases 1 and 2 and the middle phase, and the outer phase pressure. The top-right section displays counters that record frequencies of three droplet generation modes, Jet (jetting), STLD (satellite droplet), and MTC (multicore droplet).

**2. Fine-tuning of YOLOv10n object detection model**

**
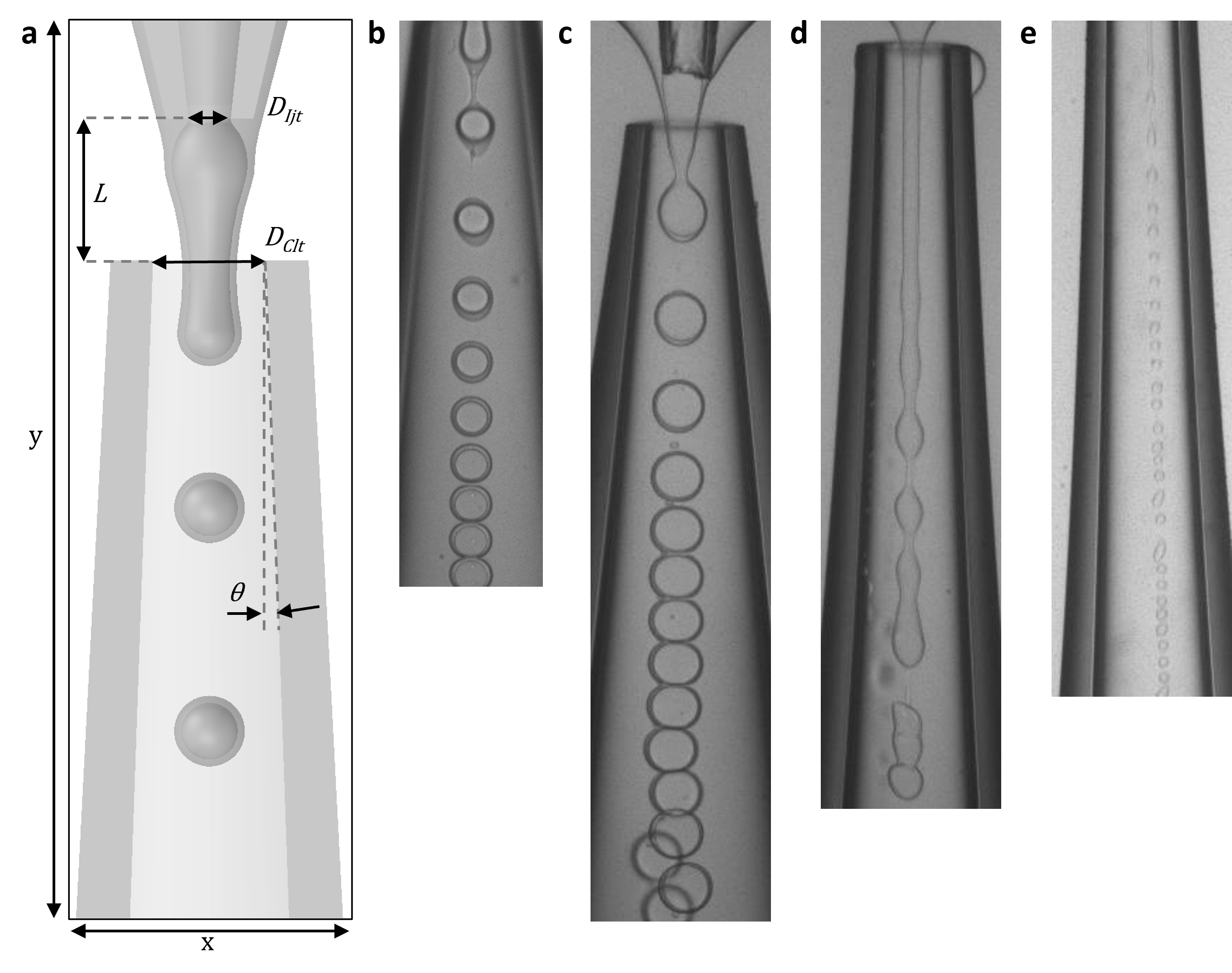
**

**Figure S2.** Types of droplet generation images constructing the dataset. (a) Schematic example of a droplet generation image. Geometrical parameters, *D_ijt_*, *D_Clt_*, *L*, and *θ* represent the inner diameter of the injection and the collection nozzles, the distance between the tips of these two capillary nozzles, and the tapered angle, respectively. x and y denote the horizontal and vertical pixel dimensions of the droplet generation image. (b-e) Examples of droplet generation images are collected using three different droplet generators, each with distinct geometries, and cropped to different size: (b) *D_Ijt_* = 45.6, *D_Clt_* = 125.1, *L*=111.1 μm, *θ*=4.4 °, and dimensions of x=100, and y=400 pixels; (c) *D_Ijt_* = 47.6, *D_Clt_* = 99.2, *L*=99.2 μm, *θ*=4.4 °, and dimensions of x=128, and y=640 pixels; (d) *D_Ijt_* = 26, *D_Clt_* = 64, *L*=103 μm, *θ*=1.8 °, and dimensions of x=128, and y=560 pixels; (e) *D_Ijt_* = 26, *D_Clt_* = 64, *L*=103 μm, *θ*=1.8 °, and dimensions of x=128, and y=480 pixels. The relative sizes of the displayed images match the relative sizes of the original images.

**
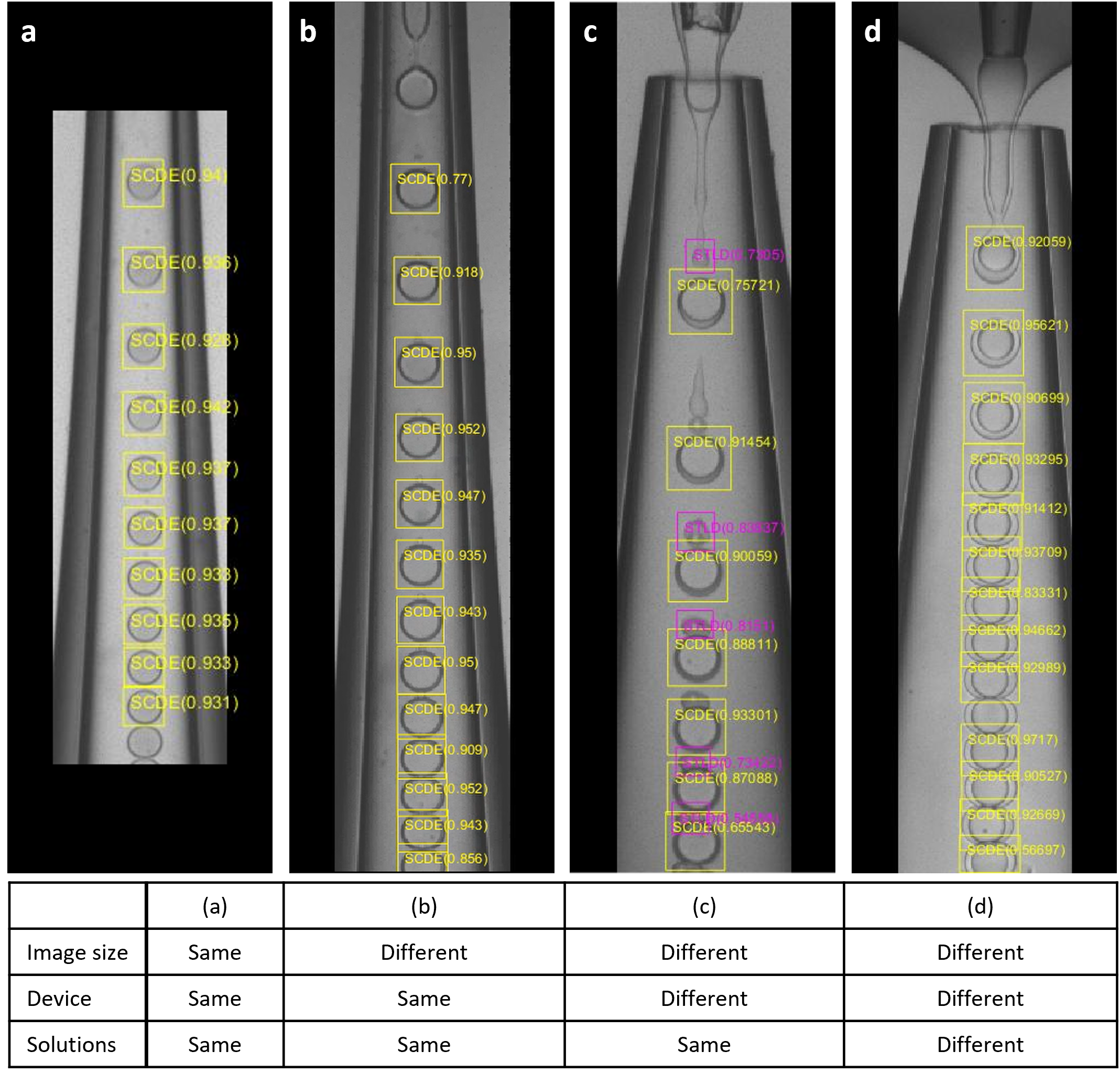
**

**Figure S3.** Object detection of droplet generation under various conditions. Detection results are overlaid on droplet generation images obtained from different experiments. (a) Detection results for an image obtained from an experiment where the droplet generator geometry, image size (128 × 480 pixels), and solutions for droplet generation are consistent with those used in this study. (b-d) Detection results for images obtained under varying experimental conditions: (b) an image cropped to a larger dimension (128 × 640 pixels); (c) an image with a different size (128 × 640 pixels) obtained from an experiment using a droplet generator with different geometry; (d) an image with a different size (128 × 640 pixels) from an experiment using a modified droplet generator geometry and 2.3% (w/w) PLGA in dichloromethane as the middle phase. The inset table specifies whether the conditions of droplet generation images match (“Same”) or differ (“Different”) from those used in this study. Displayed droplet generation images were not used to train the object detection model.


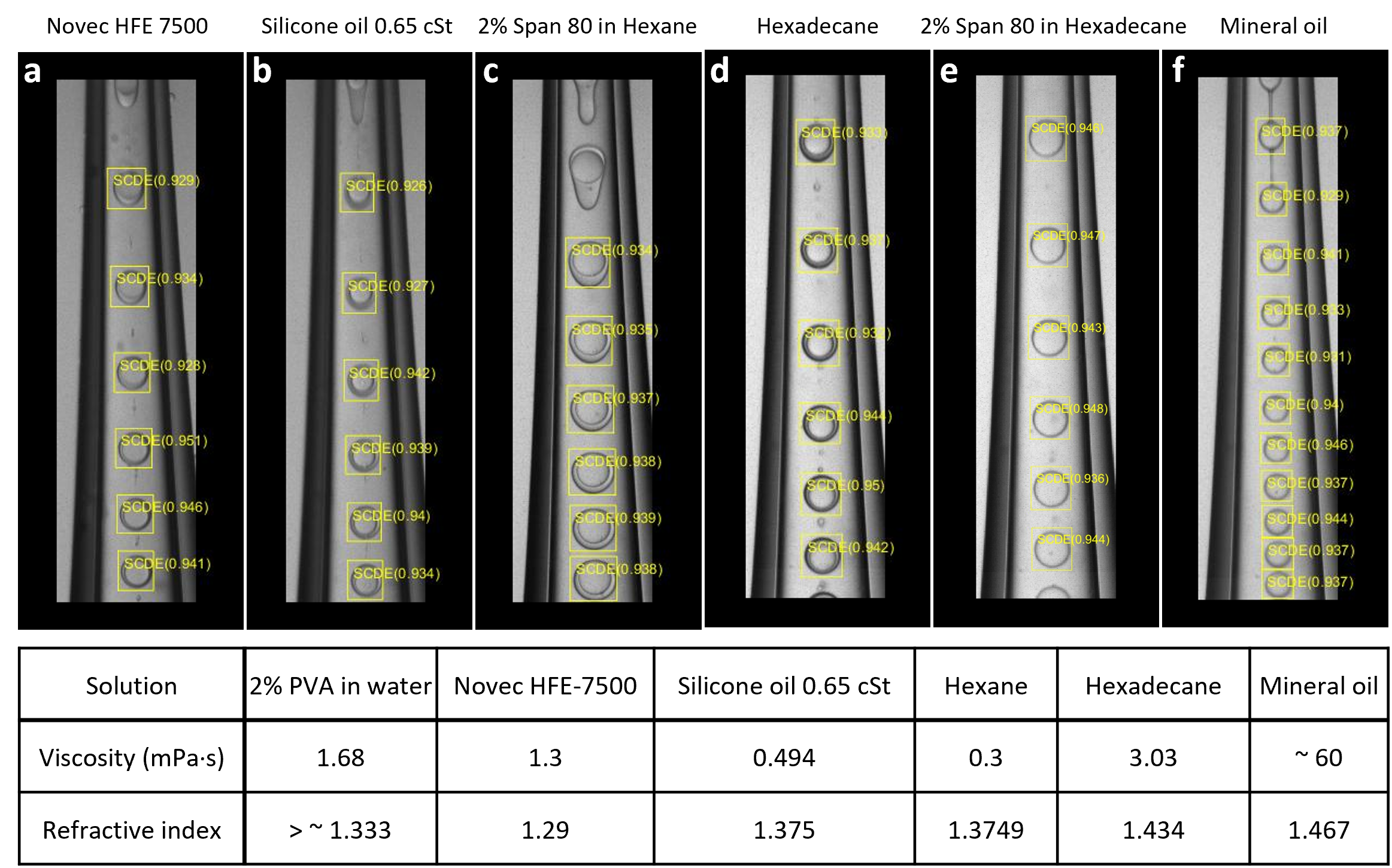


**Figure S4.** Object detection of droplet generation with various solution combinations. The inner and the outer phases consist of a 2% (w/v) PVA aqueous solution. Double emulsion droplets are generated with various middle phase solutions with a wide range of viscosities and refractive indices: (a) fluorocarbon oil (Novec HFE-7500), (b) low viscous silicone oil, (c) hexane with Span 80, (d and e) hexadecane (d) with and (e) without surfactant, and (f) mineral oil.

**3. Comparison of computer vision methods: classification and object detection**

**Table S1.** Comparison of training results using the YOLOv10n object detection model and the InceptionV3 classification model.

| Type | Name | Train | Score |
| --- | --- | --- | --- |
| Object detection | YOLOv10n | Epoch 259, best at Epoch 159 | mAP@50 = 0.9682, mAP@50-95 = 0.7457 |
| Classification | InceptionV3 | Epoch 12 | Accuracy_train=0.9935, Accuracy_valid=0.9656 |

Both computer vision models are fine-tuned using the same set of droplet generation images. In a dataset for training the classification model, the image classes are defined using the same criteria as those used to determine droplet generation modes from the object detection results in the ADLib generation program (Figure S6), ensuring that both datasets have the same ground truth. The mAP@50 and mAP@50-95 stands for the mean average precision scores of the fine-tuned object detection model, calculated when the intersection of union (IoU) threshold is 0.5 and it is between 0.5 to 0.95, respectively. The accuracy_train and accuracy_valid represent the ratio of the correct predictions over total predictions for the training and validation datasets, respectively.

**
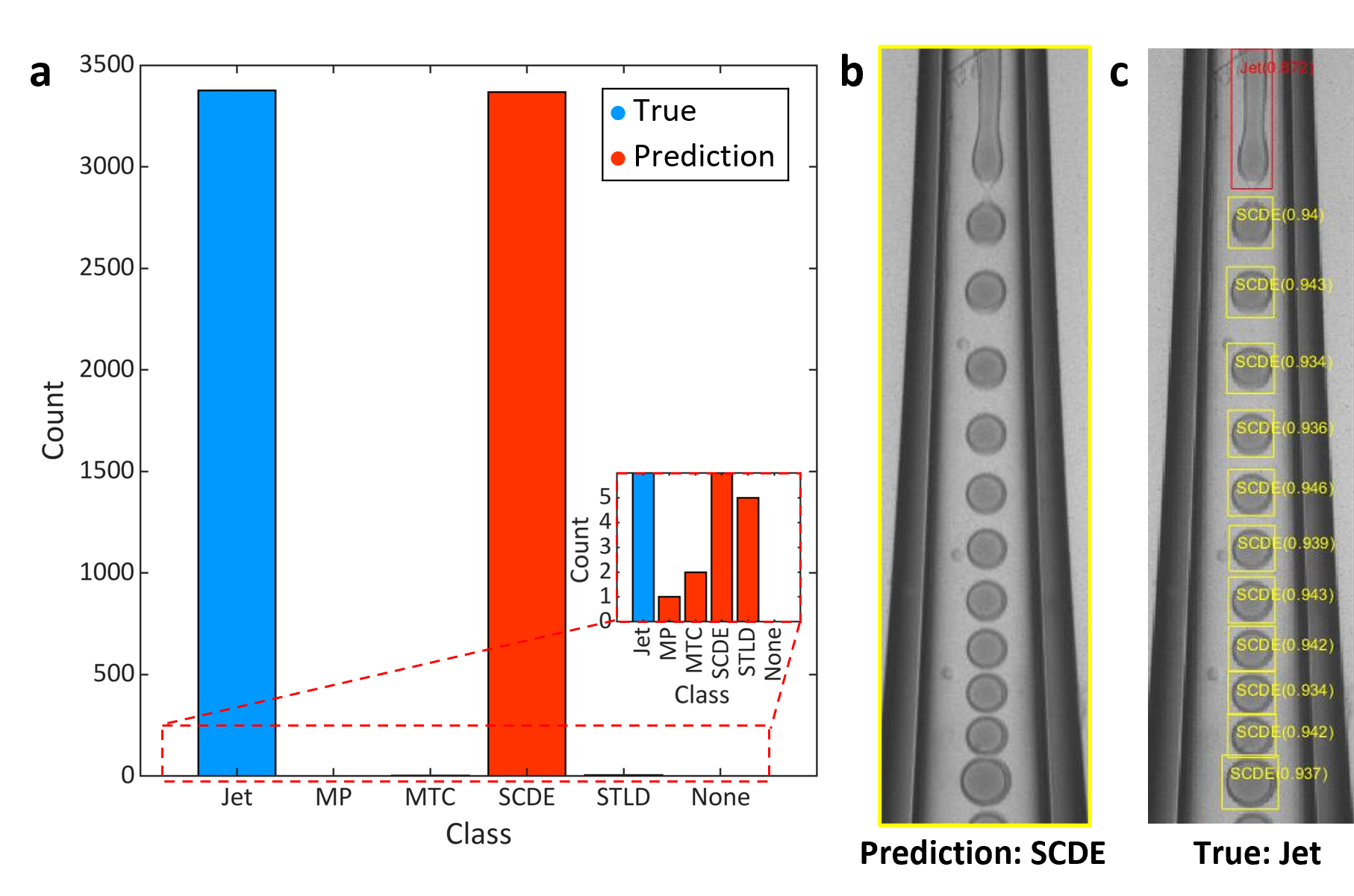
**

**Figure S5.** Comparison of prediction results of the YOLOv10n object detection and the InceptionV3 classification model. (a) Predicted classes of new droplet generation images using the fine-tuned classification model. The true class values are ‘Jet’, consistent with predictions made by the droplet library generation program using object detection results. The inset plot displays the predicted classes on a smaller y-axis scale. The total number of test images is 3,357. (b and c) Prediction results for a droplet generation image using (b) the object detection and (c) the classification model.

**4. Decision-making algorithm for selective collection of single-core double emulsion (SCDE) and SCDE droplet generation mode recovery**

**
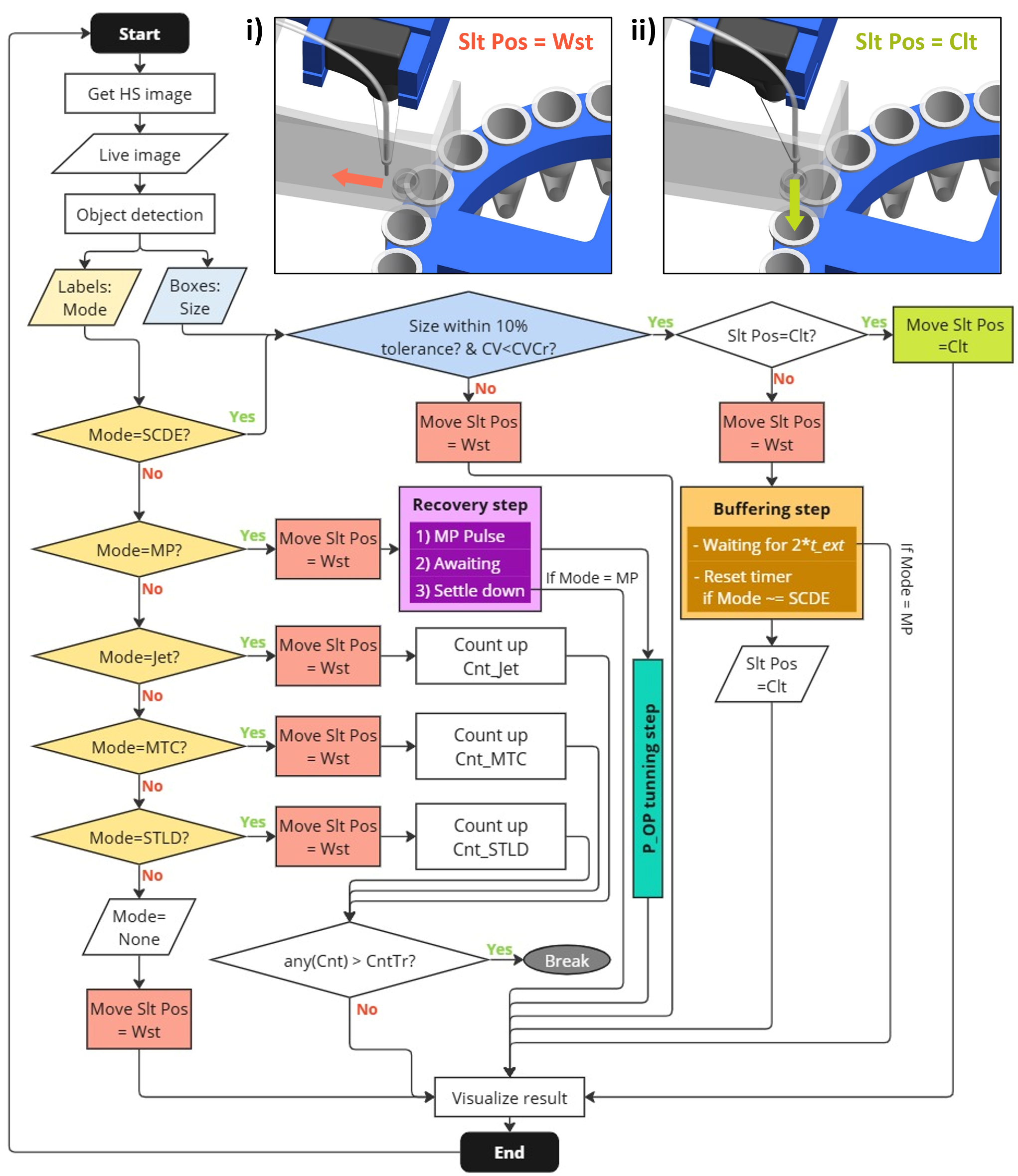
**

**Figure S6.** Flowchart of decision-making algorithm for selective collection of single-core double emulsions. Rectangles, parallelograms, diamonds, and oval represent task processing, information processing, decision-making, and loop escape blocks, respectively. Arrows indicate the flow directions. Abbreviations on the flowchart are defined following: HS Image (high-speed camera image), SCDE (single-core double emulsion), MP (middle phase only), Jet (jetting), MTC: (multicore droplets), STLD (satellite droplets), Slt Pos (product selector position), Clt (collection), Wst (waste), CV (coefficient of variation), CVCr (critical threshold of CV ), Cnt (undesirable mode counter), CntTr (counter threshold), and *t_ext* (exit time). (i and ii) Inset images display the product selector position for i) waste and ii) collection.

The ADLib generator includes a product selector and a decision-making algorithm to selectively collect SCDE droplets. The product selector, controlled by a servomotor, positions the outlet tubing. When the position of it is set to ‘Collection’, the outlet tubing is moved to align with a slit, allowing droplets to be collected into a collection vessel (Figure S6 i). When it set to ‘Waste’, the outlet tubing is moved such that it is not aligned with the slit, directing by-products to a waste vessel (Figure S6 ii).

The flowchart shows the sequence of processes in the detection and decision step for selective collection of SCDE. The program begins by obtaining a droplet generation image from the high-speed camera. The fine-tunned YOLOv10n object detection model analyzes the image providing a class label and bounding box information for detected objects. Based on the labels, the program identifies the droplet generation mode. The library generator collects SCDE droplets only if droplet generation is in the SCDE mode, the difference in approximated droplet size between the current and previous image is within a 10% tolerance, the coefficient of variation (CV) of droplet size is below the critical threshold of 7%, and the previous position of the product selector is set to ‘Collection’. Otherwise, it adjusts the product selector position to ‘Waste’.

When SCDE droplets that meet the size and uniformity criteria are detected, the program initiates a buffering step, allowing out-of-spec droplets to flow out of the device and the tubing. The buffering step is set to twice the time it takes for droplets to exit through the outlet (*t_ext_* = 9.6 sec). After buffering, the program updates the product selector status to “Collection” without physically moving it, for starting or resuming SCDE droplet collection from the next image. If other modes are detected during buffering, the program resets the timer. If MP mode is detected, the buffering is terminated, and the recovery step is triggered.

The recovery step activates whenever MP mode is detected. To restore the SCDE mode, the system delivers a pulse to the middle phase flow: the flow rate briefly increases to 100 mL/hr for 0.1 seconds then restores it to the original value. The program then waits three seconds for the microfluidic system to response. The program then allows the fluidic system to settle down for a few additional seconds (15 sec). If the compound jet is not restored, the settle down is terminated, and the program reinitiates the recovery step. This procedure is repeated until a compound coaxial water-in-oil jet is recovered. After the settle down step, the program compares the droplet size with the target size and adjusts the outer phase pressure as needed (*P_OP_* tuning step). The collection of SCDE droplet then resumes after a buffering step.

The other three modes can be caused temporarily by disturbances under flow rate settings that generate SCDE droplets. In most cases, the system will spontaneously return to SCDE mode without any intervention. However, if the source of the disturbance persists, or if the flow rate setting is unsuitable for SCDE generation, these modes may occur frequently. Each time one of these modes is detected, the program logs the information to a corresponding counter (Cnt_jet, Cnt_MTC, and Cnt_STLD). If one of these counters is over a threshold (e.g. 50 times), the library generator escapes the loop and advances to the next flow rate setting.

After completing these steps, the program displays the detection and decision results on the droplet generation image, enabling the user to monitor the current situation in real time. The program repeats this process until the specified amount of SCDE droplets is collected.

**Table S2.** Statistics of middle phase only (MP) mode occurrence in automated 5 × 5 double emulsion droplet generation experiments. The experiments were conducted sequentially from Case 1 to Case 10.

| Case | Experiment time (sec) | Frequency of MP mode | Time of the first MP mode detection (sec) |
| --- | --- | --- | --- |
| 1 | 6,838 | 8 | 364.14 |
| 2 | 7,092 | 12 | 28.25 |
| 3 | 10,171 | 44 | 164.29 |
| 4 | 7,889 | 13 | 109.44 |
| 5 | 11,900 | 95 | 9.82 |
| 6 | 13,965 | 0 | - |
| 7 | 9,888 | 3 | 4,181.64 |
| 8 | 10,365 | 11 | 474.33 |
| 9 | 8,216 | 7 | 2,046.05 |
| 10 | 8,253 | 2 | 4,640.38 |
| SUM | 94,577 | 195 | - |
| Average | 9,458±2,138 | 20±28 | 1,335.37±1,749.73 |
| Min / Max | 6,838 / 13,965 | 0 / 95 | 9.82 / 4,640.38 |


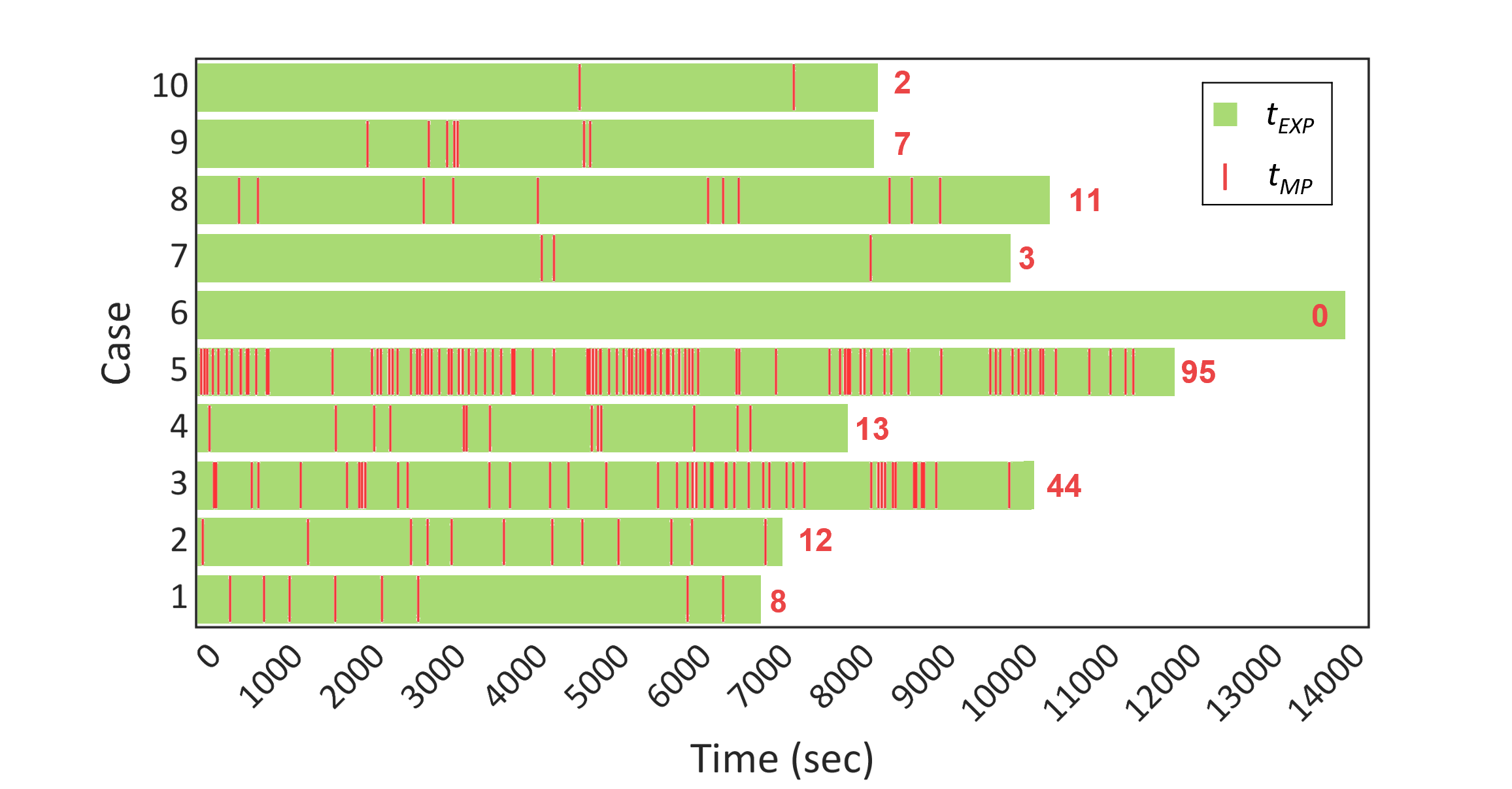


**Figure S7** Temporal frequency of middle phase only (MP) mode occurrence in automated 5 × 5 double emulsion droplet generation experiments. Each case represents the result of an experiment conducted on a different day without any human intervention. The green horizontal bars indicate a total experimental time (*t_EXP_*), while the vertical red lines mark instances when the ADLib program detects the MP mode (*t_MP_*), where the inner phase deviates from the middle phase and no double emulsions are generated. The numbers on the right of the bars indicate the frequency of MP mode occurrences. The experiments were conducted sequentially from Case 1 to Case 10.

Table S2 and Figure S7 statistically demonstrate the significance of MP mode. The data presented are from automated 5 × 5 droplet library generation experiments without human intervention. While experimental conditions such as droplet collection volume, target droplet dimensions, target concentrations, and flow rate settings varied slightly across trials, in all cases, the system successfully generated 25 distinct double emulsions by systematically adjusting flow rate ratios between the middle phase and the inner phases (*Q_MP_*/*Q_IP_* and *Q_IP2_*/*Q_IP1_*). Over the course of ten experiments using our ADLib system, MP mode was observed 195 times during a total experimental duration of 94,577 seconds (26 hr 16 min 17 sec).

The frequency of MP mode occurrence varied significantly, with an average of 20 ± 28 times per experiment, a minimum of 0 occurrences, and a maximum of 95 occurrences in ten automated experiments, with the duration of each experiment ranging from 6,838 seconds (1 hr 53 min 58 sec) to 13,965 seoncds (3 hr 52 min 45 sec). Similarly, the time to the first detection of the MP mode (excluding the cases where the MP mode did not occur) exhibited high variability, with an average of 1,335.37 ± 1,749.73 seconds, a minimum of 9.82 seconds, and a maximum of 4,640.38 seconds. These results indicate that the MP mode arises unpredictably and remains a critical challenge in double emuldion generation.

**
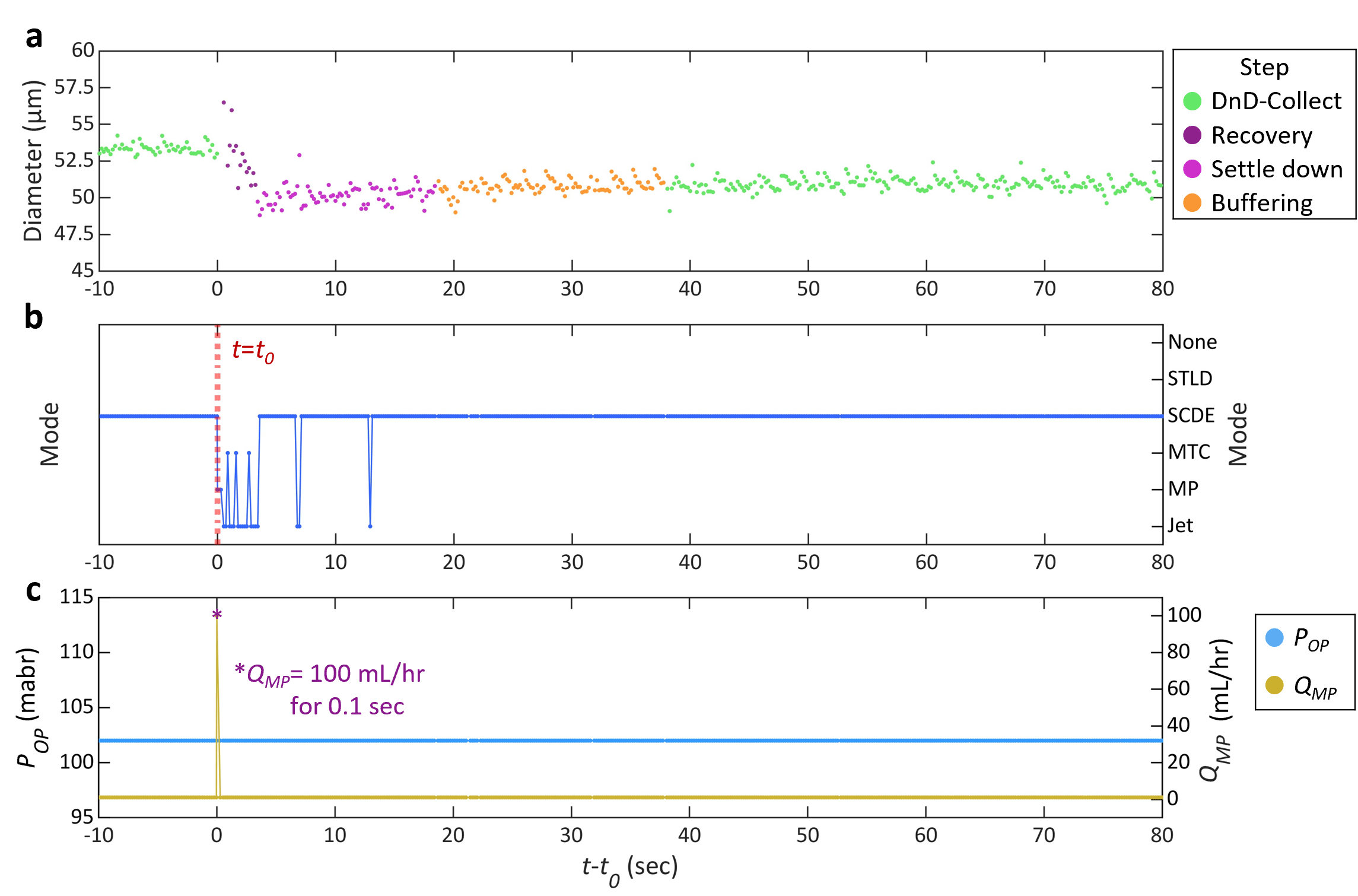
Figure S8.** Single-core double emulsion generation recovery process without “*P_OP_* tuning” step. Temporal changes in (a) the outer diameter of double emulsion droplet, (b) the detected droplet generation mode, and (c) the outer phase pressure (*P_OP_*) and the middle phase flow rate (*Q_MP_*) during the SCDE recovery process. Flow rates for the inner phase 1 and 2, and the middle phase were 0.97, 0.49, and 1.04 mL/hr, respectively. At time *t_0_*, the middle phase only (MP) mode is detected.

**5. Droplet size regulation by feedback control**

**
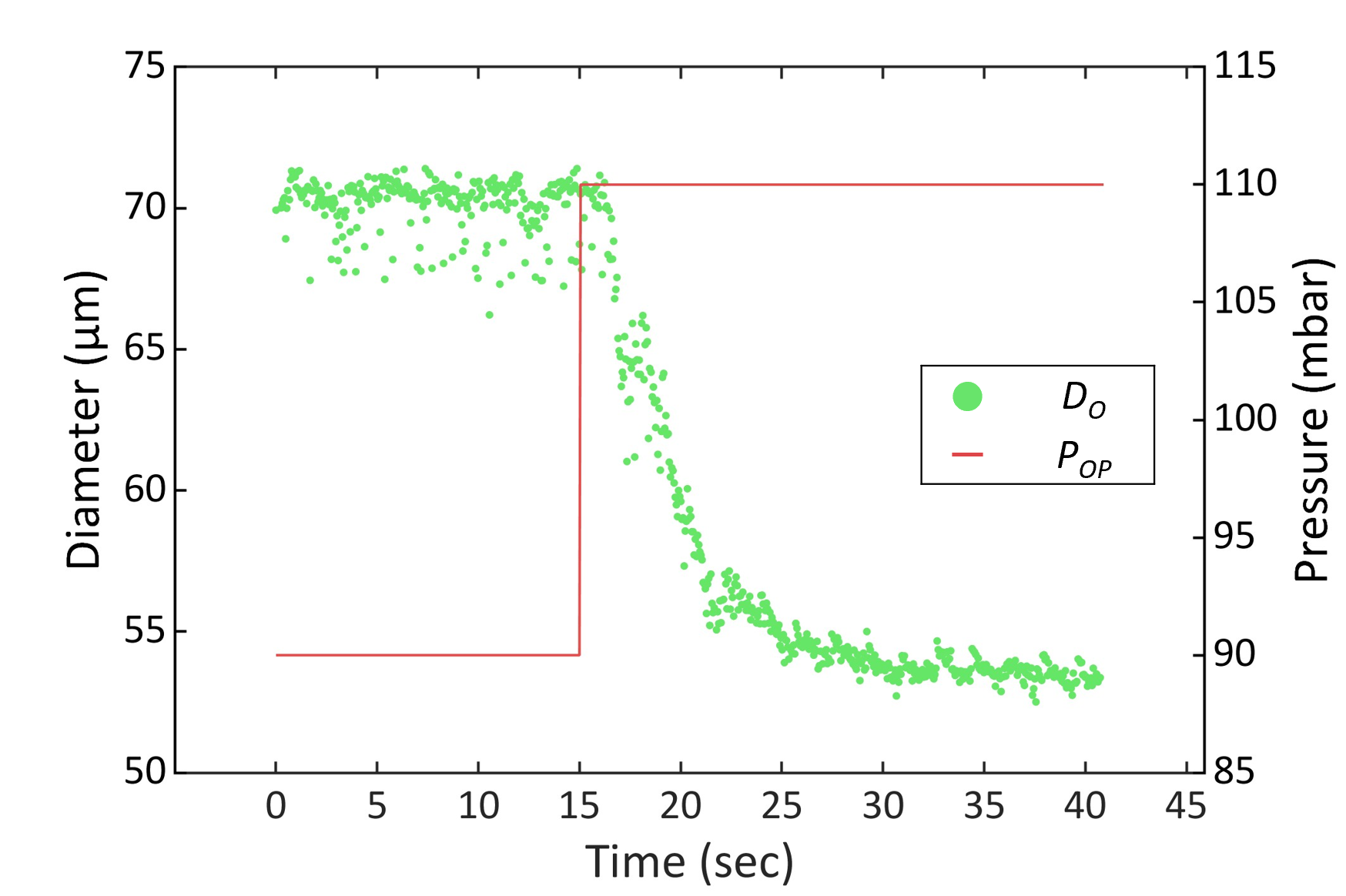
**

**Figure S9.** Response of the outer diameter (*D_O_*) of double emulsion droplets to changes in the outer phase pressure (*P_OP_*).

The type of controller and PID parameters are determined by analyzing the response of the droplet diameter (*D_O_*) to a step input of the outer phase pressure (*P_OP_*). A proportional (P) controller is selected due to its sufficiently fast response and negligible steady-state error. Additionally, its simple structure offers greater robustness compared to other controller types (PI or PID). Based on the Ziegler-Nichols method, the proportional gain (*K_P_*) is calculated to be -1.76. However, in this study, *K_P_*​ is set to -1 to match the minimum input resolution of the pressure controller (1 mbar, or 0.1 kPa), allowing for more precise control over the droplet size.


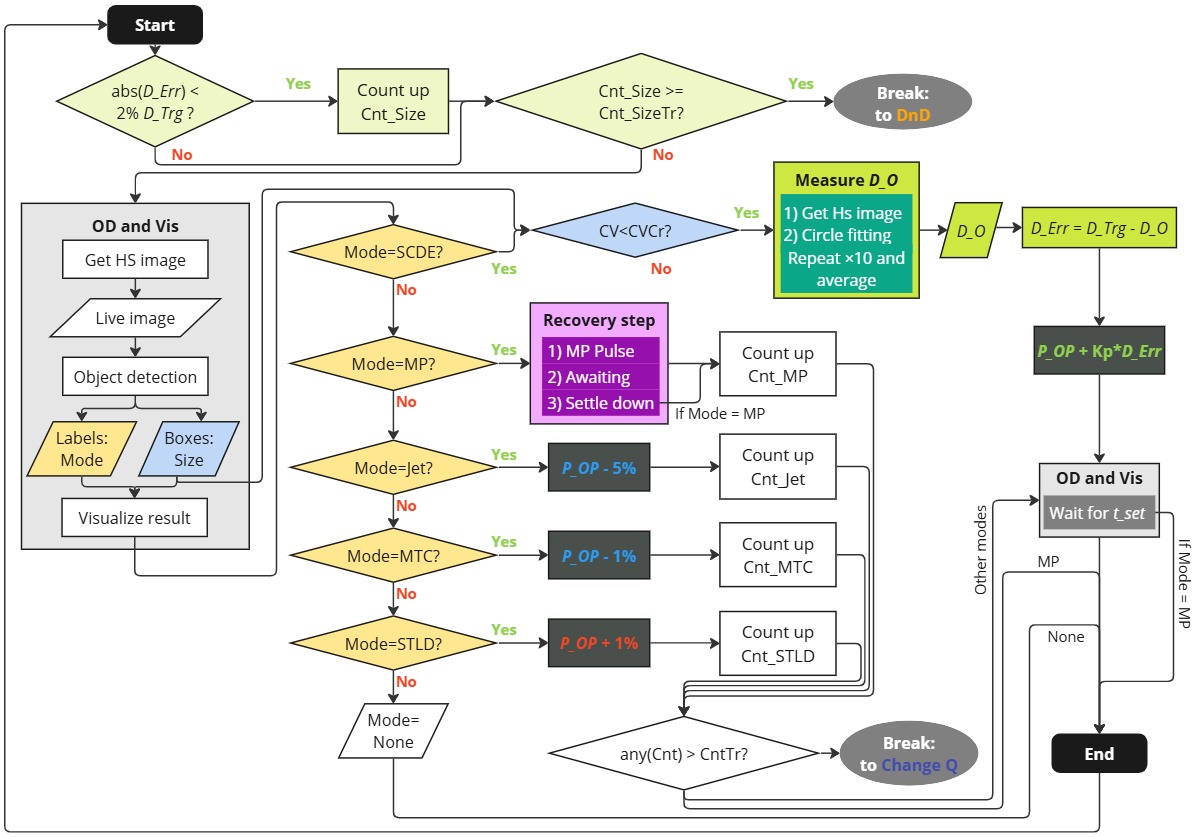


**Figure S10.** Flowchart of the feedback control algorithm for adjusting double emulsion droplet size and droplet generation mode. Rectangles, parallelograms, diamonds, and oval represent task processing, information processing, decision-making, and loop escape blocks, respectively. Arrows indicate the flow directions. Abbreviations on the flowchart are defined as following: HS Image (high-speed camera image), SCDE (single-core double emulsion), MP (middle phase only), Jet (jetting), MTC: (multicore droplets), STLD (satellite droplets), *D_O* (outer diameter of the double emulsion droplet) *D_Trg* (target *D_O*), *D_Err* (error of *D_O*), *K_P_* (proportional gain), *P_OP* (pressure of the outer phase), *t_set* (settling time of droplet generator after *P_OP_* change), Cnt_Size (loop terminate condition counter), Cnt_SizeTr (threshold value of Cnt_Size), DnD (detection and decision step for selective collection of SCDE), CV (coefficient of variation), CVCr (critical threshold of CV ), Cnt_Mode (unstable mode counter), CntTr (threshold value of Cnt_Mode), Change Q (change flow rate setting step).

The flowchart outlines the sequence of processes in the feedback control loop for adjusting the outer diameter of the double emulsion droplet (*D_O_*). In this loop, the pressure of the outer phase (*P_OP_*) is adjusted until the termination conditions are met. If the detected mode is SCDE and the coefficient of variation of the approximated droplet sizes is less than 7%, the program measures *D_O_*, which is determined as the average of ten measurements using the circle-fitting method. The error (*D_Err_*) is calculated as target diameter (*D_Trg_*) minus *D_O_*. The *P_OP_* is then updated by the proportional controller as *P_OP_* + *K_P_*·*D_Err_*. The program waits 5 sec, which we define as the settling time (*t_set_*) to allow the pressure controller and the fluid system to respond to the updated *P_OP_*. The value for *t_set_* depends on the performance of the pressure controller. If *D_Err_* is less than 2 % of *D_Trg_* (approximately 1 μm in this study), the loop break condition counter (Cnt_Size) is incremented. When Cnt_Size exceeds the threshold value (Cnt_SizeTr=2), the feedback control loop terminates, and the program proceeds to the detection and decision (DnD) step for the selective collection of SCDE with a size of *D_Trg_*.

If an undesirable droplet generation mode is detected, the program adjusts *P_OP_* or initiates the recovery step to restore the SCDE mode. When the Jet or MTC modes are detected, *P_OP_*, which is proportional to the outer phase flow rate, is decreased by 5% or 1%, respectively, to reduce the Capillary number of the outer phase (*Ca_O_*).^[1, 2]^ Conversely, when the STLD modes is detected, the *P_OP_* is increases by 1% to raise *Ca_O_*.^[3]^ Since the *P_OP_* condition for the SCDE mode falls between undesirable modes, extreme changes in *P_OP_* are avoided. If the current flow rate settings for the inner phase and middle phase are unsuitable for the SCDE mode, undesirable modes are frequently observed. Each time such a mode is detected, the undesirable mode counter (Cnt_Mode) is incremented. If Cnt_Mode exceeds the threshold value (CntTr), the program exits the loop and proceeds to the next flow rate setting (Change Q step). This mechanism prevents the program from becoming trapped in an infinite loop.


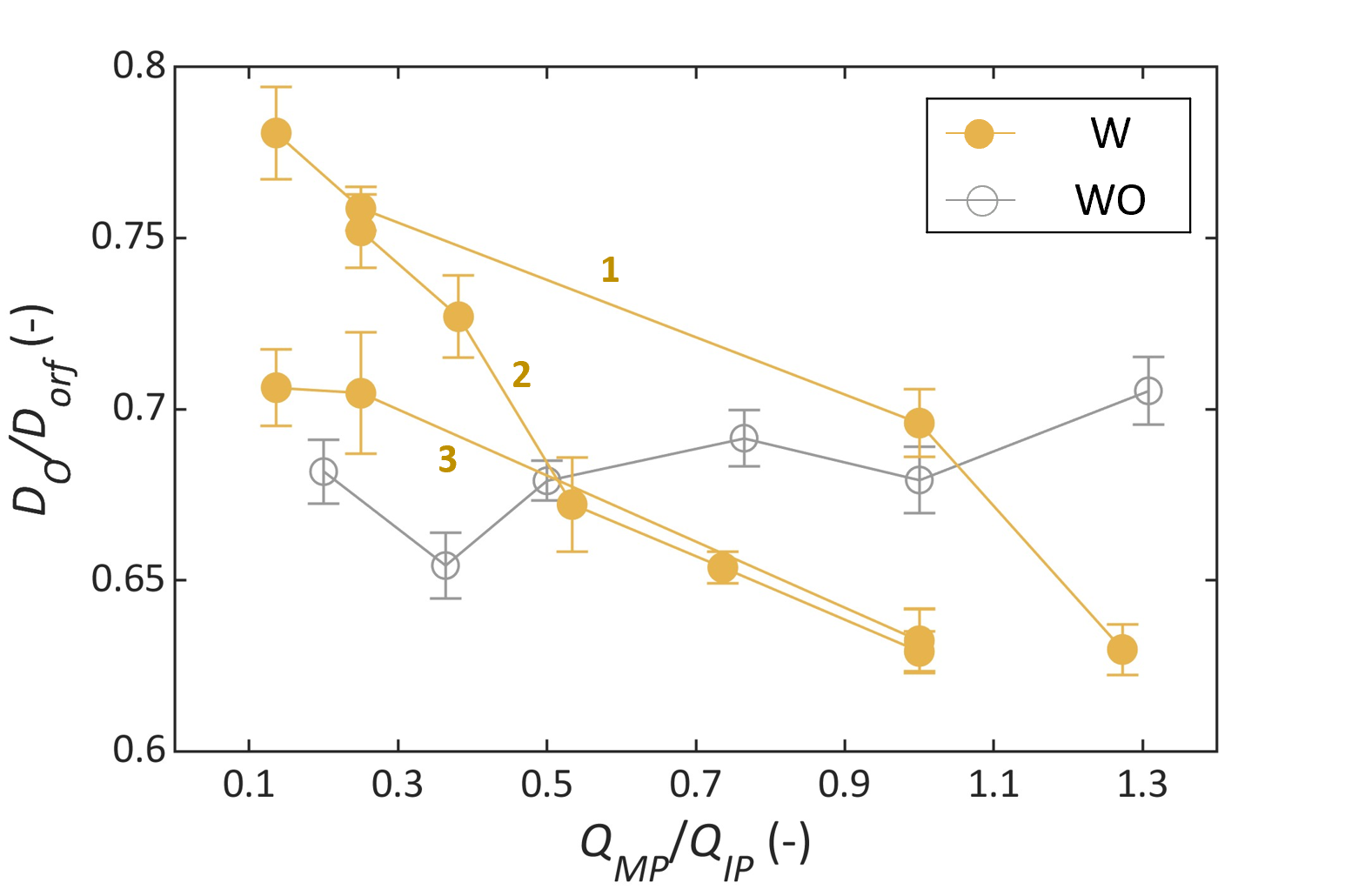


**Figure S11.** Effect of the flow rate ratio of the middle phase to the inner phase and the presence of surfactant on the outer diameter of the double emulsion droplet. The outer diameter (*D_O_*) is nondimensionalized by dividing it by the inner diameter of collection capillary (*D_orf_*). *Q_IP_* and *Q_MP_* represent the flow rate of the inner phase and the middle phase, respectively. “W” (with) and “WO” (without) denote the presence of surfactant in the middle phase fluid. The dots connected by lines represent measurements taken when *Q_DP_* and *Q_OP_* are held constantly. The error bars represent the standard deviation. For the experiment without a surfactant (W/O), the flow rate settings are: *Q_DP_* = 3 mL/hr and *Q_OP_* = 25 mL/hr. For the experiments with a surfactant (W), the flow rate and the pressure settings are as follows: 1) *Q_DP_* = 2.5 mL/hr and *Q_OP_* = 15 mL/hr; 2) *Q_DP_* = 2.5mL and *P_OP_* = 87 mbar; 3) *Q_DP_* = 2.5 mL/hr and *Q_OP_* = 17 mL/hr

For simplicity, we assume that the double emulsion generation system in the SCDE mode behaves similarly to a single droplet generation system, treating the inner and middle phases as a single dispersed phase. Under constant *Q_DP_* and *Q_OP_*, the dimensionless numbers affecting *D_O_* are the viscosity ratio ($\lambda={\mu_{eff}}/{\mu_{OP}}$) and the Capillary number (${Ca}_{OP}={\mu_{OP}U}/{\gamma_{eff}}$). Here, $\mu_{OP}$, $U$, $\mu_{eff}$, and $\gamma_{eff}$ indicate the dynamic viscosity of the outer phase fluid, fluid velocity at the droplet generation junction, the effective viscosity of the dispersed phase, and the effective interfacial tension between the dispersed and the outer phases. The viscosity of the inner and outer phase fluid is 1.68 mPa·s,^[4]^ while the viscosity of the middle phase with and without the surfactant is around 1.6 and 1.3 mPa·s, respectively.^[5]^

As shown in Figure S11, *D_O_* slightly increases with *Q_MP_*/*Q_IP_* in the absence of the surfactant. This increase corresponds to a decrease in $\mu_{eff}$, interpreted as a reduction in $\lambda$, meaning that more tangential viscous drag is dissipated into the internal flow of the dispersed phase, leading to larger droplets.^[6]^ Conversely, when the surfactant is present in the middle phase, *D_O_* decreases significantly as *Q_MP_*/*Q_IP_* increases, despite $\mu_{MP}$ being slightly lower than $\mu_{IP}$. According to the result of a numerical simulation, when the viscosity of the inner and middle phase is identical and surfactants are not present, *D_O_* remains constant regardless of variation in *Q_MP_*/*Q_IP_*.^[7]^ This variation of *D_O_* by *Q_MP_*/*Q_IP_* can be interpreted as following: as the middle phase layer becomes thinner, i.e. as *Q_MP_*/*Q_IP_* decreases, the local surfactant coverage at the interface decreases, increasing $\gamma_{eff}$ and decreasing ${Ca}_{OP}$, resulting in larger droplets at lower frequency.

**6. Calculation of flow rates using user input**

**
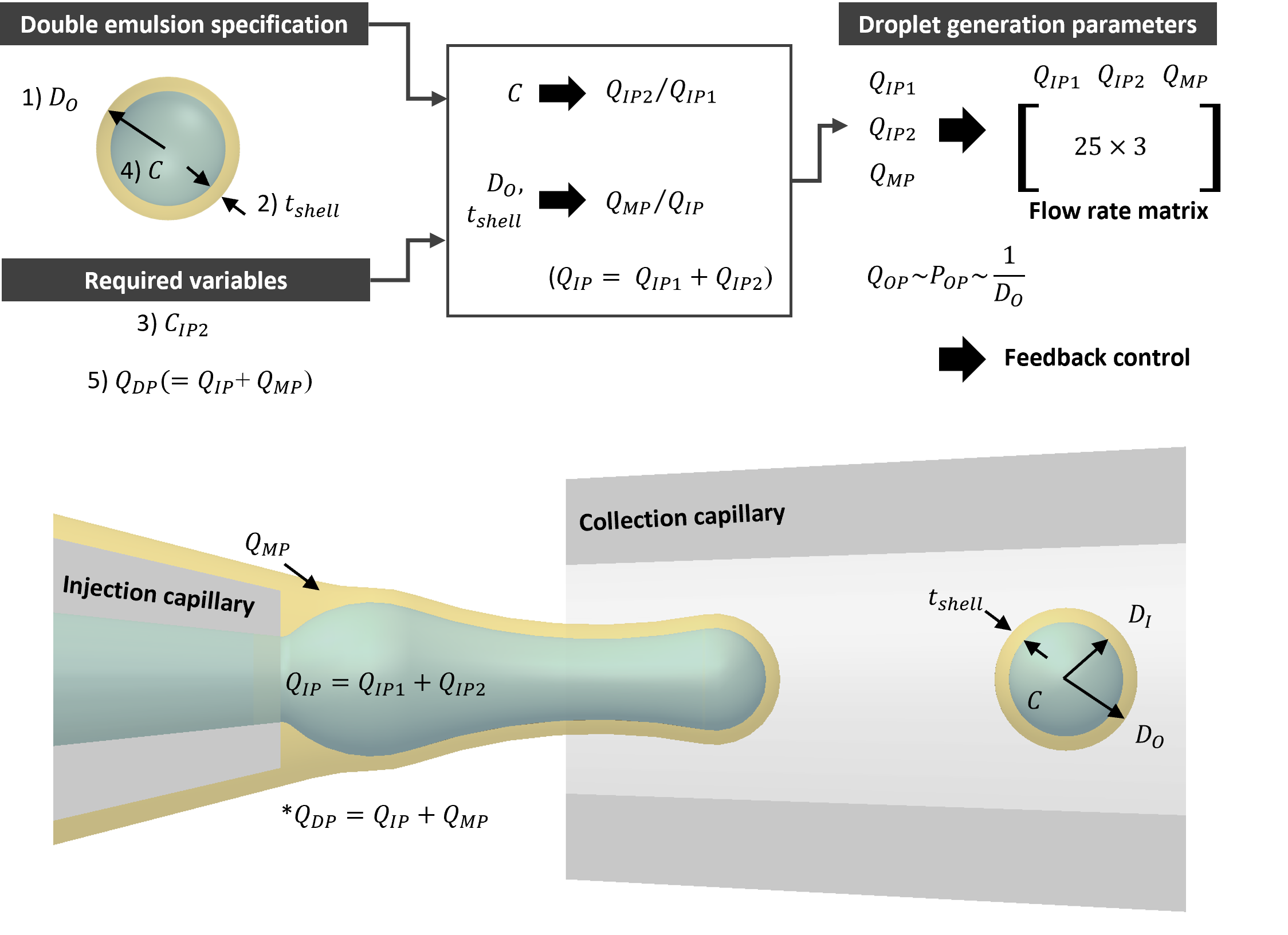
**

**Figure S12.** Generation of flow rate matrix based on the user input parameters.

The ADLib program requires five user-defined input parameters: outer diameter (*D_O_*), five shell thickness values (*t_shell_*), the solute concentration of the inner phase 2 (*C_IP2_*), either a range (minimum and maximum concentrations) or five discrete concentration values (*C*), and the dispersed phase flow rate (*Q_DP_*), as shown in Figure S1a and b. The flow rate ratio of the middle phase to the inner phase (*Q_MP_*/*Q_IP_*) is derived from *D_O_* and *t_shell_*, as defined in Equation S1. The inner and middle phase flow rates are calculated using (*Q_MP_*/*Q_IP_*) and *Q_DP_*, as described in Equation S2. Since *t_shell_* is a 5 × 1 vector, the resulting flow rate matrix for *Q_IP_* and *Q_MP_* forms a 5 × 2 matrix. Further, *Q_IP_* is partitioned into *Q_IP1_* and *Q_IP2_* using the values of *C* and *C_IP2_*, as described in Equation S3. Given the five target concentrations, the resulting flow rate matrix for *Q_IP1_*, *Q_IP2_*, and *Q_MP_* is constructed as a 25 × 3 matrix. The ADLib system generates 25 droplets sequentially by varying the flow conditions according to the computed values in this flow rate matrix.

$\frac{Q_{MP}}{Q_{IP}}=\frac{V_{MP}}{V_{IP}}=\left( \frac{D_{O}}{D_{O}-2t_{shell}} \right)^{3}-1$ (S1)

$Q_{IP}=\left( \frac{D_{O}-2t_{shell}}{D_{O}} \right)^{3}Q_{DP}, Q_{MP}=\left[ 1-\left( \frac{D_{o}-2t_{shell}}{D_{O}} \right)^{3} \right]Q_{DP}$ (S2)

$C=C_{IP2}\frac{Q_{IP2}}{Q_{IP}}$ (S3)

**7. Response of the double emulsion generator to flow rate changes**

In this study, the response of the double emulsion generator to dispersed phase flow rate changes is experimentally measured. 10 mL plastic syringe (BD Plastipak) was used for the middle phase, while syringes for the inner phases were varied. The syringes were driven by PHD ULTRA syringe pumps (Harvard Apparatus) and connected to the microfluidic device via fluorinated ethylene propylene (FEP) tubing (1 mm OD × 0.5 mm ID, IDEX). Flow rates of the middle phase (*Q_MP_*) and the inner phase (*Q_IP_*= *Q_IP1_* + *Q_IP2_*) were kept constant as 0.5 mL/hr and 2 mL/hr, respectively, while the flow rate ratio of the inner phases (*Q_IP2_*/*Q_IP1_*) varied from 0.3/1.7 to 1.7/0.3. The results are depicted in Figure S13 and Table S3.


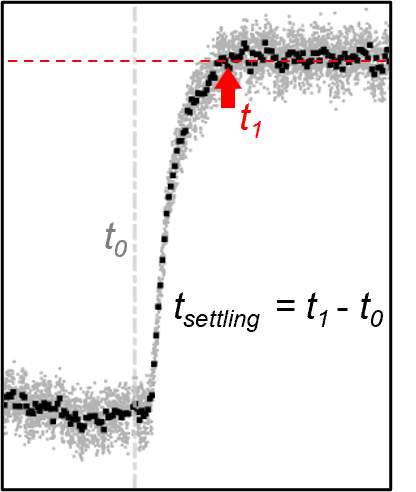
**Table S3.** Effect of syringe type on the double emulsion generator response to the inner phase flow changes. The settling time (*t_settling_*) represents the duration required for the gray value to reach a plateau following a flow rate change (*t_1_* - *t_0_*).

| Syringe type | BD plastic 10mL | BD plastic 3mL | Hamilton glass  2.5 mL |
| --- | --- | --- | --- |
| Response | *t_settling_* (sec) | *t_settling_* | *t_settling_* |
| Average | 213.80±41.27 | 184.07±18.87 | 220.14±33.76 |
| Min | 150.03 | 161.93 | 178.90 |
| Max | 268.53 | 215.99 | 277.31 |


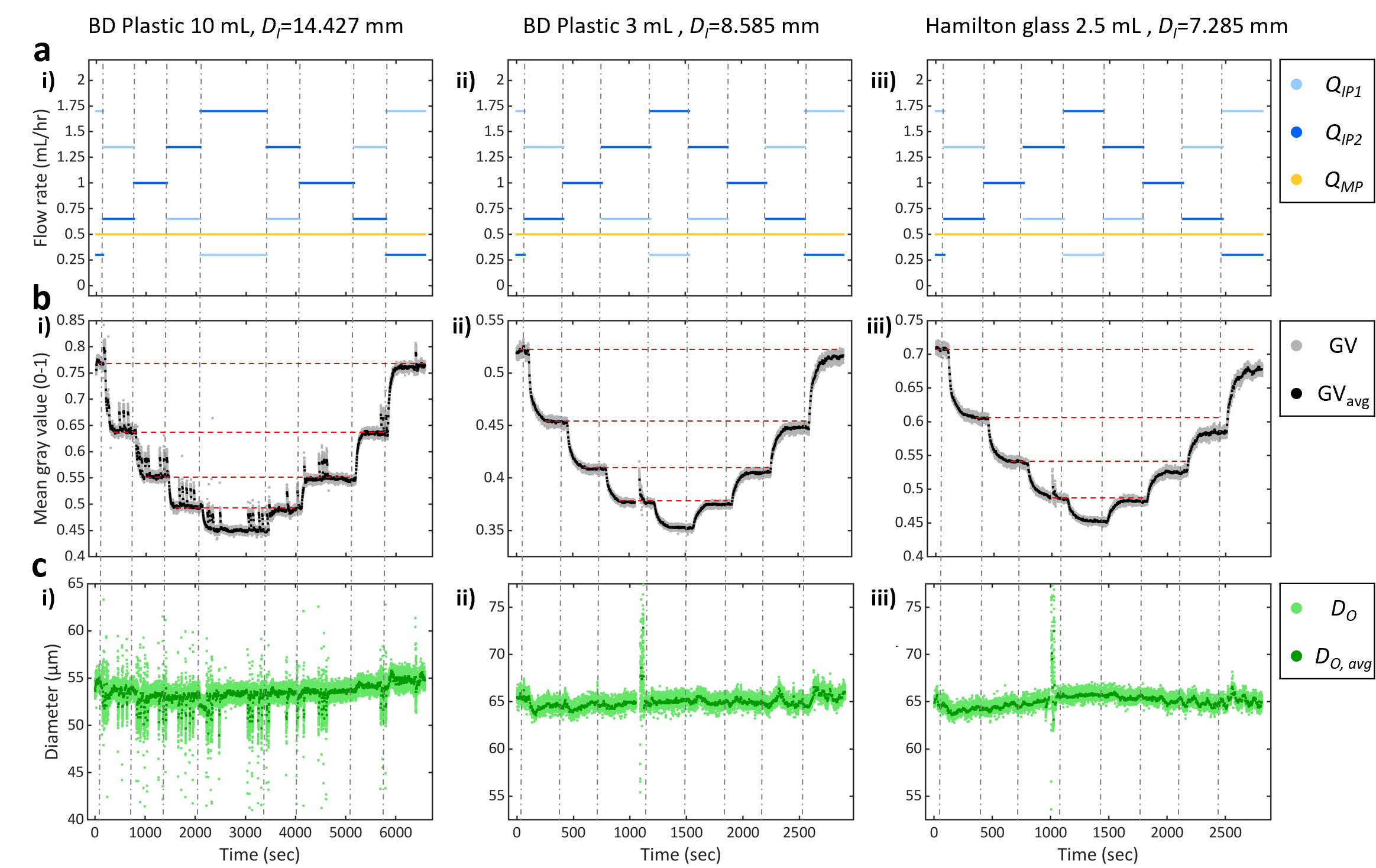


**Figure S13.** Response of the double emulsion generator to the inner phase flow rate changes based on syringe type. Temporal changes of (a) flow rates of the inner phase 1 and 2, and middle phase, (b) gray value, and (c) outer diameter (*D_O_*) of double emulsion droplets captured from high-speed images. The average gray value (GV_avg_) and diameter (*D_O, avg_*) are calculated as the moving average of 25 neighboring values. Drastic changes in GV and *D* indicate the occurrence of the MP mode and subsequent recovery of the SCDE mode. The light gray dash-dot lines denote instances when *Q_IP2_* /*Q_IP1_* changes, and the red dashed lines represent the average gray value of the droplet as *Q_IP2_*/*Q_IP1_* increases. The experiments used different syringes for the two inner phases: i) BD plastic 10mL, ii) BD plastic 3mL, and iii) Hamilton glass 2.5mL. *D_I_* represents the inner diameter of the syringe.

The settling time (*t_settling_*) is found to be significantly longer than the entrance time —the time it takes for the inner phases to reach the droplet generation junction — and exhibited high variability with a large standard deviation. This extended settling time is primarily caused by the inherent delay between adjusting the syringe pump flow rates and the corresponding changes in droplet generation within the microfluidic device. The delay arises because the system requires time for pressure inside the syringe to equilibrate. Additionally, the high fluidic resistance within the microfluidic system, including the glass capillary double emulsion generator and the inner phase micromixer, contributes to this delay, as pressure stabilization is necessary before an actual flow rate change occurs.

Furthermore, the compliance of the syringe and tubing increase latency, as the system requires time to develop sufficient pressure to adjust the actual flow rate. Using syringes with smaller inner diameters helps to reduce latency; replacing 10 mL plastic syringes with 3 mL ones decreases *t_settling_* by 19%. However, the more rigid glass syringe exhibits the longest *t_settling_* with significant discrepancies in the gray value of the droplets, which represents the concentration of Evans blue, under identical *Q_IP1_*/*Q_IP2_* conditions. This inconsistency is attributed to fluid leakage past the syringe piston during the experiment. Given its large volume and high reliability, 10 mL plastic syringe was selected for use in this study, with a flow rate change time of 270 seconds for the inner phase.

The flow rate change time for *Q_MP_* and *Q_IP_* is measured as 90 seconds using a similar approach. Even when *Q_IP2_*/*Q_IP1_* ratio remains constant, the change in *Q_MP_*/*Q_IP_* inevitably influences *Q_IP2_* and *Q_IP1_*, contributing to additional latency. However, when only a single inner phase was used, *t_settling_* can be significantly reduced as shown in Figure S14.


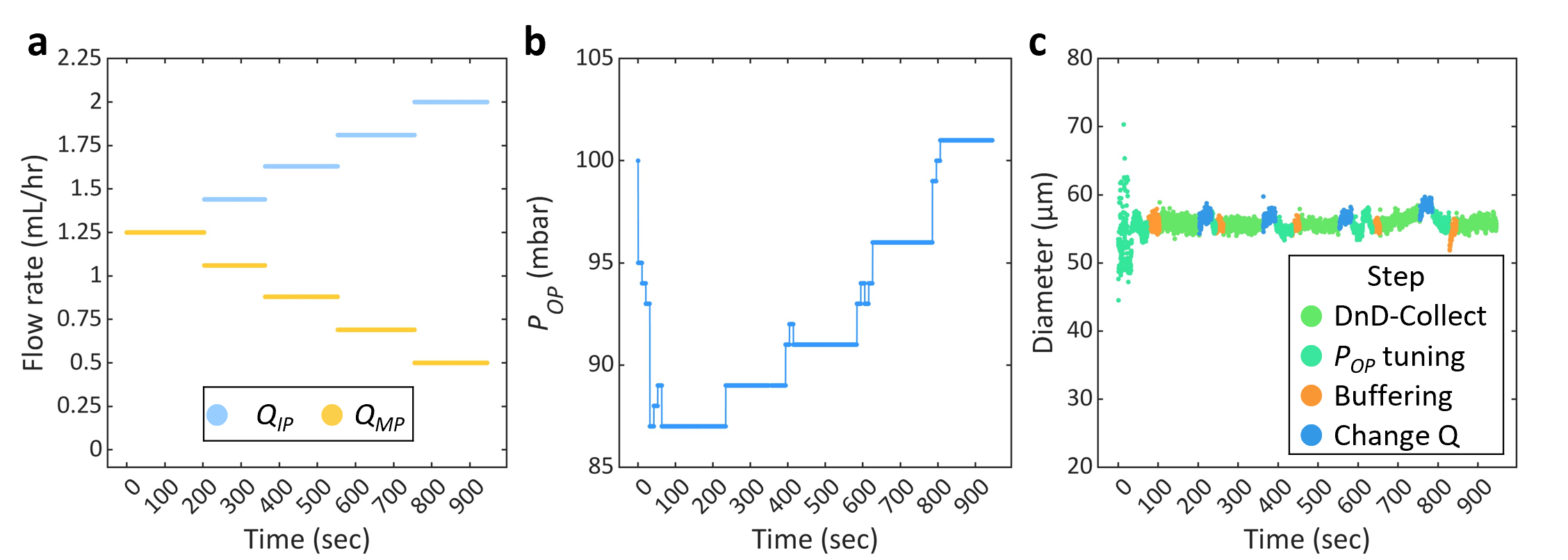


**Figure S14.** Automated double emulsion droplet generation with a single inner phase solution and a flow rate changing time of 30 seconds. Temporal variation of (a) flow rates for the inner phase and middle phase, (b) the outer phase pressure as adjusted by feedback control, and (c) outer diameter (*D_O_*), with different colors representing each step the an automated droplet library generator (ADLib) program.

**8. Supporting videos**

**Video S1.** Failure of double emulsion generation.

In this video, MP mode occurs spontaneously, leading to failure in double emulsion generation. The droplet generator used has the following geometry: *D_Ijt_* = 61.5, *D_Clt_* = 123, *L* = 89.3 μm and, *θ*=2.3 °. Flow rates for the inner, middle, and outer phases were set to 2, 0.75, and 40 mL/hr, respectively.

**Video S2.** Effect of product selector movement in droplet generation.

The top section of the video shows droplet generation while the product selector alternates between "collection" and "waste" positions every 0.5 seconds. The bottom section displays droplet generation without product selector movement. Flow rates for inner phase 1 and the middle phase were set to 2 and 0.5 mL/hr, respectively, with an outer phase pressure of 100 mbar.

**Video S3.** Single-core double emulsion generation recovery process.

This video shows droplet generation with overlaid detection results. MP mode occurs spontaneously. Flow rates for inner phase 1 and 2, and the middle phase were set to 1.7, 0.3, and 0.5 mL/hr, respectively.

**Video S4.** Demonstration of graphical user interface 1.

This video demonstrates the initiation of automated double emulsion droplet library generation using the GUI. The experiment shown in the video is based on the same user input parameters as described in Figures 5, 6, and Video S6. This version of the GUI accepts minimum and maximum concentration values and calculates five linearly spaced concentrations between them.

**Video S5.** Demonstration of graphical user interface 2.

This video demonstrates the initiation of automated double emulsion droplet library generation using the GUI when the initial droplet generation mode is MP. In this version of the GUI, users can input five specific concentration values.

**Video S6.** Automated generation of double emulsion droplet library.

This time-lapse video shows the generation process of a 5 × 5 droplet library. Results from this experiment are presented in Figures 5 and 6 in the main text.

**SI Reference**

[1] Utada, A. S.; Lorenceau, E.; Link, D. R.; Kaplan, P. D.; Stone, H. A.; Weitz, D., *Science* **2005,** *308* (5721), 537-541.

[2] Nabavi, S. A.; Vladisavljević, G. T.; Bandulasena, M. V.; Arjmandi-Tash, O.; Manović, V., *Journal of colloid and interface science* **2017,** *505*, 315-324.

[3] Zhu, P.; Kong, T.; Kang, Z.; Tian, X.; Wang, L., *Scientific reports* **2015,** *5* (1), 11102.

[4] Scholes, P.; Coombes, A.; Illum, L.; Daviz, S.; Vert, M.; Davies, M., *Journal of controlled release* **1993,** *25* (1-2), 145-153.

[5] Calhoun, S. G.; Brower, K. K.; Suja, V. C.; Kim, G.; Wang, N.; McCully, A. L.; Kusumaatmaja, H.; Fuller, G. G.; Fordyce, P. M., *Lab on a Chip* **2022,** *22* (12), 2315-2330.

[6] Dewandre, A.; Rivero-Rodriguez, J.; Vitry, Y.; Sobac, B.; Scheid, B., *Scientific reports* **2020,** *10* (1), 21616.

[7] Yang, Z.; Ma, X.; Wang, S.; Liu, D., *Chemical Engineering Science* **2022,** *255*, 117683.
